# Importance of structural hinderance in performance–stability equilibrium of organic photovoltaics

**DOI:** 10.1038/s41467-022-33754-3

**Published:** 2022-10-08

**Authors:** Baobing Fan, Wei Gao, Xuanhao Wu, Xinxin Xia, Yue Wu, Francis R. Lin, Qunping Fan, Xinhui Lu, Wen Jung Li, Wei Ma, Alex K.-Y. Jen

**Affiliations:** 1 Department of Chemistry, City University of Hong Kong, Kowloon, Hong Kong 999077 China; 2 Hong Kong Institute for Clean Energy, City University of Hong Kong, Kowloon, Hong Kong 999077 China; 3 Department of Materials Science and Engineering, City University of Hong Kong, Kowloon, Hong Kong 999077 China; 4 State Key Laboratory for Mechanical Behavior of Materials, Xi’an Jiaotong University, Xi’an, 710049 P. R. China; 5 Department of Physics, The Chinese University of Hong Kong, New Territories, Hong Kong, 999077 China; 6 Department of Mechanical Engineering, City University of Hong Kong, Hong Kong, 999077 China; 7 Department of Materials Science & Engineering, University of Washington, Seattle, WA 98195 USA

**Keywords:** Solar cells, Solar cells

## Abstract

Power conversion efficiency and long-term stability are two critical metrics for evaluating the commercial potential of organic photovoltaics. Although the field has witnessed a rapid progress of efficiency towards 19%, the intrinsic trade-off between efficiency and stability is still a challenging issue for bulk-heterojunction cells due to the very delicate crystallization dynamics of organic species. Herein, we developed a class of non-fullerene acceptors with varied side groups as an alternative to aliphatic chains. Among them, the acceptors with conjugated side groups show larger side-group torsion and more twisted backbone, however, they can deliver an efficiency as high as 18.3% in xylene-processed cells, which is among the highest values reported for non-halogenated solvent processed cells. Meanwhile, decent thermal/photo stability is realized for these acceptors containing conjugated side groups. Through the investigation of the geometry**–**performance**–**stability relationship, we highlight the importance of side-group steric hinderance of acceptors in achieving combined high-performance, stable, and eco-friendly organic photovoltaics.

## Abstract (web summary)

The intrinsic trade-off between efficiency and stability is a challenging issue for organic photovoltaics. Here, the authors develop non-fullerene acceptors with tailored side-group hindrance and backbone planarity and realise efficiency of 18.3% and good stability for xylene-processed solar cells

## Introduction

Organic photovoltaics (OPV) is quite promising for portable electricity generation in areas from flexible/wearable electronics to indoor applications^1,2^. Similar to the requirements for traditional photovoltaic (PV) technologies, OPV needs to satisfy the critical power conversion efficiency (PCE) and long-term stability metrics in order to be considered for their commercialization^3,4^. The replacement of fullerene derivatives by non-fullerene acceptors (NFAs) has helped ascend the PCE of single-junction OPV to beyond 18%^5–11^, closing the gap with other PV technologies^12^. Meanwhile, this strategy also improves the OPV stability by overcoming the molecular diffusion and aggregation of spherical fullerene acceptors^13–15^. However, the trade-off between PCE and stability still remains in bulk-heterojunction (BHJ) OPV, because of the meta-stable morphology in the active layer. The originally well phase-separated nanoscale channels in active layer for hole- and electron-transport/collection tend to form excessive scale aggregates during long-term aging, which is detrimental to the device efficiency and lifetime^16,17^.

Significant efforts have been devoted to improving the long-term stability of OPV, e.g., through cross-linking to freeze the bulk morphology of active layer^18,19^. This strategy provides an extrinsic solution for overcoming the morphologic instabilities of OPV. However, the introduction of foreign species and the use of ultraviolet light for curing inevitably compromise the device performance. To improve the intrinsic stability of OPV without sacrificing the device efficiency, the focus should return to the most essential chemical structures of NFAs.

In addition to the photo-oxidation and photocatalyzed nucleophilic reactions that tend to break the NFA’s conjugation to degrade their light absorbing capabilities^20–23^, the relationship between their chemical structures and the morphological evolution under external stresses also needs to be concerned. Thermal stress induced instability is a very challenging problem for OPV^24^, because the best-performing BHJ devices are often achieved when the active layer morphology is in the thermodynamically non-equilibrated state^24^, which needs to be maintained during the whole thermal aging process. This can be potentially accomplished by tailoring the backbone planarity and side-group hinderance of NFAs, since these structure features are directly related to the crystallization and diffusion abilities of NFAs and their interactions with polymer matrix^25,26^.

However, the tailoring of NFA structures for improving their thermal stability should not compromise the PCE, and it would be ideal if the balance between stability and PCE can be achieved by using lower toxicity processing solvents. To process high-performance OPV using eco-friendly solvents such as xylene, NFAs are usually decorated with long aliphatic side chains to ensure good solubility in these solvents without really affecting the active layer morphology^27–30^. However, the increased aliphatic chain length often results in less densely packed NFAs and larger local free volume, which increases the segmental motion^31–33^. To avoid this destabilizing factor, the side groups of NFAs should also be subtly tuned besides the backbone to mitigate the free volume-caused molecule diffusion.

**Fig. 1:**
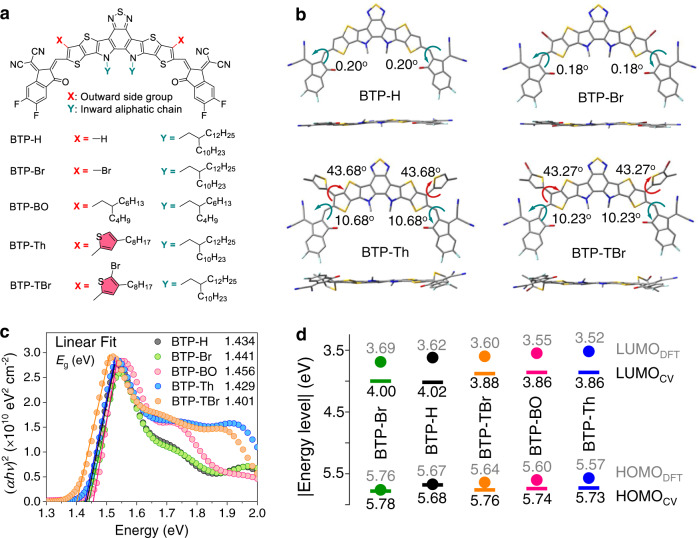
Molecular structure/geometry and photoelectric properties. **a** Chemical structure of acceptors with varied side groups. **b** Optimized molecular geometry for acceptors calculated from DFT; the red and green arrows indicate the outward side group and terminal group torsions, respectively. **c** Optical bandgap of neat acceptors determined from Tauc plots. **d** Energy level alignments determined from both DFT and CV methods.

In this work, a series of A-DA′D-A-type NFAs with varied side groups on the outward positions of π-core are developed (Fig. 1a). We demonstrate that the side-group torsion and backbone planarity of NFAs can be readily adjusted by tailoring their outward side groups. Specifically, non-substituted and brominated NFAs show rather planar backbone while thiophene-substituted ones have twisted skeleton with large side-group torsions. Owing to the narrow bandgap and moderate crystallization, the twisted acceptors perform well in xylene-processed solar cells, with the best PCE reaching 18.3%, among the highest efficiencies reported for non-halogenated solvent processed OPV. Meantime, a decent thermal/photo-thermal stability is realized for these acceptors with large structural steric hinderance. By investigating the relationship between structure/geometry and morphology/performance evolution under thermal stress, we highlight that a suitable degree of structural hinderance for NFAs is important for achieving the performance–stability equilibrium in OPV.

## Results

### Molecular structure and geometry

Five acceptors with significantly different side groups were synthesized based on a general procedure described in the Supplementary Information. BPT-H and BPT-Br were prepared from the starting compound of non-substituted and brominated thieno[3,2-*b*]thiophene, respectively (Supplementary Fig. 1 and 2). The intermediate of BTP-Br π-core was used to couple with non-substituted and brominated thiophene (Th and TBr) by using Stille coupling reaction before condensing with the 2-(5,6-difluoro-3-oxo-2,3-dihydro-1*H*-inden-1-ylidene)malononitrile (IC-2F) terminal group, finally generating the target acceptors of BTP-Th and BTP-TBr, respectively (Supplementary Fig. 3). The structure of these acceptors was characterized by proton nuclear magnetic resonance (Supplementary Fig. 4–7) and matrix-assisted laser desorption/ionization time-of-flight mass spectrometry (Supplementary Fig. 8), confirming the accuracy and high purity of these materials.

Density functional theory (DFT) calculations were performed to reveal obviously different optimal molecular geometries for these acceptors, where an extra acceptor BTP-BO with commonly used aliphatic chain was included for comparison (Fig. 1a)^34^. The side views of the molecular geometries show a very planar backbone for BTP-H and BTP-Br with a dihedral angle as small as 0.20^o^ and 0.18^o^ between the fused core and IC-2F wings (Fig. 1b), while that for BTP-BO is 3.14^o^ (Supplementary Fig. 9a). This might be caused by the steric hinderance of the outward 2-butyloctyl chains on BTP-BO. When the side groups were changed to the thiophene moieties, the steric hinderance further increases the backbone dihedral to 10.68^o^ and 10.23^o^ for BTP-Th and BTP-TBr, respectively (Fig. 1b). Besides, these three acceptors show large side-group torsion angles (> 43^o^), which could inhibit the potential excessive aggregation of NFAs induced by strong backbone interactions under external stresses.

### Optical properties and energetics

The light absorption profiles of BTP-H and BTP-Br were characterized to reveal the electronic effect of bromo group. From the solution absorption depicted in the Supplementary Fig. 10a, we note an obviously blue-shifted spectrum of BTP-Br compared to that of BTP-H. This could be attributed to the electron-withdrawing inductive effect of the bromo substituent. In solid state, this blue shift is largely mitigated (Supplementary Fig. 10b), implying it is influenced by molecular aggregation. However, BTP-Br still shows a slightly larger optical band-gap (*E*_g_) compared to that of BTP-H (1.441 vs. 1.434 eV, Fig. 1c). The situation is different when compare BTP-Th vs. BTP-TBr, where an obviously lower *E*_g_ of 1.401 eV is observed for BTP-TBr compared to 1.429 eV for the non-brominated counterpart. These results suggest there is a different electronic effect when the bromo group is on the backbone than that on the thienyl side group. When comparing the film absorption of all five acceptors, we find those with conjugated side groups show red-shifted absorption, and BTP-BO with four aliphatic side chains has the largest *E*_g_. Moreover, acceptors with larger structural torsion show enhanced shoulder peak in the high photon energy region, which is commonly observed for acceptors containing conjugated side groups^35–37^.

The DFT calculations also reveal the molecular frontier orbital distributions of these acceptors (Supplementary Fig. 12). Both the lowest unoccupied molecular orbital (LUMO) and highest occupied molecular orbital (HOMO) are affected by the outward side groups, giving gradually up-shifted energy levels for BTP-Br, BTP-H, BTP-TBr, BTP-BO, and BTP-Th (Fig. 1d). The DFT results match well with the LUMO levels determined from electrochemical cyclic voltammetry (CV, Supplementary Fig. 10c, d). Compared to BTP-H, the higher LUMO level of acceptors with bulky side groups (i.e., BTP-TBr, BTP-Th, and BTP-BO) implies a potentially higher open-circuit voltage (*V*_OC_) for solar cells based on these acceptors. These acceptors have varied HOMO levels ranging from −5.70 to −5.78 eV (Supplementary Table 1), matching well with that of the polymer donor PTzBI-*d*F (−5.59 eV, Supplementary Fig. 9b)^38^. Considering a low *E*_g_ of 1.401 eV and a relatively high LUMO energy level for BTP-TBr, it may perform well in solar cell devices.

### Performance of xylene-processed devices

Solar cells were fabricated to probe both electronic and steric effects of side groups on photovoltaic performance. Chloroform (CF) was initially used to deposit the active layers. Devices based on PTzBI-*d*F and varied acceptors show significantly different photovoltaic parameters (Supplementary Fig. 12, Supplementary Table 2). BTP-BO having the highest LUMO level yields the highest *V*_OC_ of 0.893 V in devices, while its largest *E*_g_ gives the lowest short-circuit current density (*J*_SC_) of 22.9 mA cm^−2^. Such an imbalance also exists in BTP-Br-devices, where a decent *J*_SC_ of 25.8 mA cm^−2^ is achieved but affording a very low *V*_OC_ of 0.782 V. BTP-H-devices show a relatively balanced *V*_OC_ (0.840 V) and *J*_SC_ (26.2 mA cm^−2^) but displaying a modest fill factor (FF) of 70.5%, resulting in a moderate PCE of 15.5%. This dilemma is mitigated in BTP-TBr based devices, where a decent PCE of 17.6% is achieved with balanced parameters of 0.840 V (*V*_OC_), 26.8 mA cm^−2^ (*J*_SC_), and 77.7% (FF). In comparison, BTP-Th based devices show a higher *V*_OC_ of 0.852 V and a lower *J*_SC_ of 25.1 mA cm^−2^, consistent with the shifts in *E*_g_ and LUMO level induced by the electronic effect of bromo group.

**Fig. 2:**
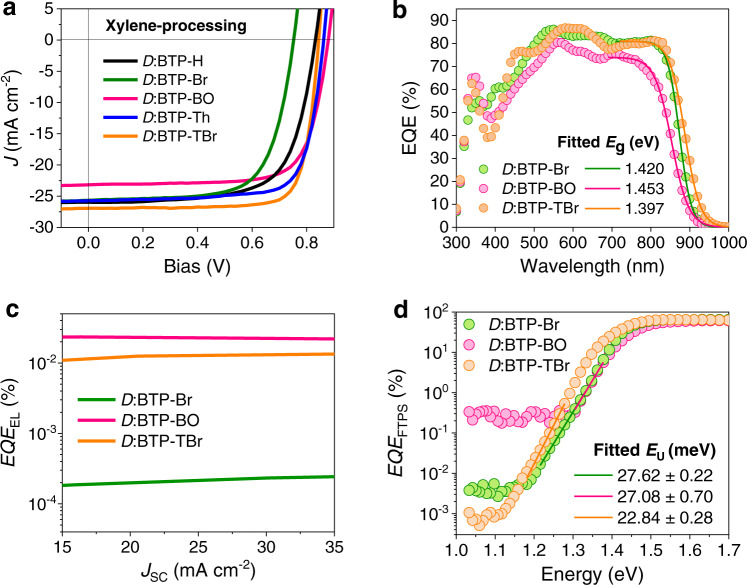
Photovoltaic performance and energetic analysis. *J* − *V* characteristic (**a**) and EQE spectra (**b**) for conventional devices processed by xylene, where *D* represents PTzBI-*d*F; the bandgap of the blends was obtained from sigmoidal fitting of EQE spectra in the low photon energy region. **c** *EQE*_EL_ for devices based on various acceptors. **d** EQE calculated from FTPS (*EQE*_FTPS_); *E*_U_ was obtained by exponential fitting of the low photon energy region of FTPS.

Since acceptors bearing long aliphatic side chains show good solubility in non-halogenated solvents, they were thus evaluated in xylene (XY)-processed devices. A comparable performance is achieved as that for CF-processed devices. Particularly, acceptors with large structural torsion perform better in XY-processed devices (Fig. 2a, Table 1). For instance, BTP-TBr-devices show simultaneously improved *J*_SC_ (26.9 mA cm^−2^) and FF (79.2%), leading to a PCE of 18.0%. The deposition of a thin layer of MgF_2_ as anti-reflection coating (ARC) on the back side of device substrate further increases the PCE to 18.3% due to improved external quantum efficiency (EQE, Supplementary Fig. 13). This is among the highest PCEs for OPV processed by non-halogenated solvent. In contrast, BTP-Th-devices exhibit a lower PCE of 16.7% due to a modest *J*_SC_ of 25.7 mA cm^−2^, which is reasonable given its relatively higher *E*_g_ and more twisted backbone.

**Table 1:** Photovoltaic parameters for xylene-processed solar cells based on polymer donor PTzBI-*d*F and various acceptors

Acceptor	*V*_OC_ (V)^a^	*J*_SC_ (mA cm^−2^)	FF (%)	PCE (%)^b^
BTP-H	0.832 (0.829 ± 0.003)	26.0 (25.3 ± 0.4)	68.4 (67.3 ± 1.6)	14.8 (14.1 ± 0.4)
BTP-Br	0.752 (0.746 ± 0.003)	25.7 (25.2 ± 0.3)	69.1 (67.8 ± 1.1)	13.3 (12.8 ± 0.3)
BTP-BO	0.880 (0.877 ± 0.004)	23.2 (22.7 ± 0.5)	73.9 (73.2 ± 0.9)	15.1 (14.6 ± 0.3)
BTP-Th	0.863 (0.864 ± 0.003)	25.7 (25.9 ± 0.2)	75.4 (74.0 ± 0.7)	16.7 (16.6 ± 0.2)
BTP-TBr	0.844 (0.839 ± 0.003)	26.9 (27.0 ± 0.3)	79.2 (78.0 ± 0.7)	18.0 (17.7 ± 0.2)
	0.845	27.5	78.8	18.3^c^

^a^The errors in the bracket are defined as standard deviation.

^b^Average of at least 16 individual devices.

^c^Anti-reflection coating (MgF_2_) of 140 nm was deposited on the back side of ITO glass.

For devices based on planar acceptors, i.e., BTP-H and BTP-Br, modest PCEs of <15% are obtained mainly due to the low FFs of 68–69%. These much lower FFs than those of devices based on twisted acceptors shows the importance of suitable structural steric hinderance of acceptors in achieving an optimal bulk morphology. Therefore, the steric effects of side groups on critical device properties and stability were thoroughly investigated. Three representative acceptors, i.e., BTP-Br, BTP-BO, and BTP-TBr, with distinctly different side-group hinderance were selected for detailed comparisons. BTP-TBr-devices show a broader EQE response than those for BTP-Br- and BTP-BO-counterparts (Fig. 2b), consistent with their band-gaps. Besides, the integrated current densities confirm a small mismatch with those obtained from current density−voltage (*J* − *V*) curves (Supplementary Fig. 14a).

### Energy losses in xylene-processed devices

*V*_OC_ losses were analyzed to elucidate the steric effects of side groups on energy losses. A high electroluminescence quantum efficiency (*EQE*_EL_) around 2 × 10^−4^ is achieved in BTP-BO- and BTP-TBr-devices, while BTP-Br-devices show a very low value of 2.18 × 10^−6^ (Fig. 2c). The non-radiative *V*_OC_ loss (*ΔV*_OC, nr_) calculated from the well-established relationship is summarized in Table 2^39^. Compared with BTP-BO-devices having a low *ΔV*_OC, nr_ of 0.216 V, the incorporation of conjugated side group slightly enlarges the *ΔV*_OC, nr_ (0.230 V for BTP-TBr-devices), while devices based on planar acceptor BTP-Br show significantly increased *ΔV*_OC, nr_ of 0.335 V. The radiative *V*_OC_ loss was also investigated to reveal other recombination loss pathways^40^. The sigmoidal fitting of EQE in low photon energy region gives the *E*_g_ of blends (Fig. 2b, Supplementary Table 3)^41^. As seen in Table 2, BTP-TBr-devices not only have a low *ΔE*_3_ (i.e., *qΔV*_OC, nr_) but also show the lowest *ΔE*_2_ (i.e., *qV*_OC, SQ_ − *qV*_OC, rad_), therefore delivering the smallest energy loss (*E*_loss_) of 0.553 eV while those for BTP-Br- and BTP-BO-devices being 0.668 and 0.573, respectively.

**Table 2:** *V*_OC_ and energy losses for xylene-processed solar cells based on PTzBI-*d*F donor

Acceptor	*E*_g_^*a*^ (eV)	*EQE*_EL_ @*J*_SC_	*ΔV*_OC, nr_^*b*^ (V)	*V*_OC, SQ_^*c*^ (V)	*V*_OC, rad_^*d*^ (V)	*ΔE*_1_^*e*^ (eV)	*ΔE*_2_^*f*^ (eV)	*ΔE*_3_^*g*^ (eV)	*E*_loss_^*h*^ (eV)
BTP-Br	1.420	2.18 × 10^−6^	0.335	1.164	1.087	0.256	0.077	0.335	0.668
BTP-BO	1.453	2.27 × 10^−4^	0.216	1.196	1.096	0.257	0.100	0.216	0.573
BTP-TBr	1.397	1.28 × 10^−4^	0.230	1.144	1.074	0.253	0.070	0.230	0.553

^*a*^*E*_g_ is determined from one-step fitting of EQE spectra from Almora’s sigmoidal function.

^*b*^*ΔV*_OC, nr_ (*T*) = − ( *k*_B_*T*/*q*) ln(*EQE*_EL_).

^*c*^*V*_OC, SQ_ is Shockley-Queisser limit of *V*_OC_.

^*d*^*V*_OC, rad_ = *V*_OC, meas_ + *ΔV*_OC,nr_, where *V*_OC, meas_ is the mesaured *V*_OC_.

^*e*^*ΔE*_1_ = *E*_g_ − *qV*_OC, SQ_.

^*f*^*ΔE*_2_ = *q*(*V*_OC, SQ_ − *V*_OC, rad_).

^*g*^*ΔE*_3_ = *qΔV*_OC, nr_.

^*h*^*E*_loss_ = *ΔE*_1_ + *ΔE*_2_ + *ΔE*_3_ = *E*_g_ − *qV*_OC, meas_.

To validate the small *E*_loss_ in BTP-TBr-devices, we performed the Fourier-transform photocurrent spectroscopy (FTPS) to sensitively detect the EQE (*EQE*_FTPS_) in the low photon energy region. Through exponential fitting of the band edge of *EQE*_FTPS_^42,43^, we attain a low Urbach energy (*E*_U_) of 22.84 ± 0.28 meV for BTP-TBr-devices, while that for BTP-Br- and BTP-BO-counterparts is 27.62 ± 0.22 and 27.08 ± 0.70, respectively (Fig. 2d, Supplementary Table 4). The much lower energetic disorder explains well the suppressed *ΔE*_2_ in the BTP-TBr-devices. The charge-transfer state energy (*E*_CT_) was examined to reveal the origin of different *ΔV*_OC, nr_ in these devices based on Marcus theory^44^. As plotted in Supplementary Fig. 15, *E*_CT_ is obtained as a cross point of the fitted para-curves, giving a value of 1.384, 1.380, and 1.346 eV for BTP-Br, BTP-BO, and BTP-TBr based cells, respectively. We find no dependence of *ΔV*_OC, nr_ on *E*_CT_ for these systems^45–47^. However, we note a broadened emission linewidth for the devices based on planar acceptors (Supplementary Fig. 14b), which is believed to stem from the increased total reorganization energy induced by the enhanced aggregation in blends^48^, validating the higher *ΔV*_OC, nr_ for these devices relative to those based on twsited accepor.

The relative dielectric constants (*ε*_r_) of acceptors and their blends were evaluated by capacitance–frequency (*C*_p_–*f*) plots (Supplementary Fig. 16). Compared with BTP-Br that has a *ε*_r_ of ~2.4, the incorporation of bulky side group increases the *ε*_r_ to ~3.1 for BTP-TBr. In the blends including PTzBI-*d*F (*ε*_r_ = ~4.0), the overall *ε*_r_ of BTP-Br-blend is promoted to 2.89 ± 0.03, respectively, while BTP-TBr-blend still shows higher *ε*_r_ of 3.01 ± 0.02. This might be due to the torsional character of the conjugated side group in BTP-TBr that can afford the reorientation of dipole moments^49^. The *ε*_r_ value of blend film can be used to quantify the binding energy of CT exciton (*E*_B_^CT^) from the equation, *E*_B_^CT^ = *q*^2^/(4π*ε*_0_*ε*_r_*r*_0_), where *r*_0_ is the average distance for electron–hole separation distance^50^. Clearly, the enhanced *ε*_r_ in BTP-TBr-blend corresponds to a reduced *E*_B_^CT^, which could facilitate the exciton dissociation and mitigate the charge recombination in solar cells. This agrees well with the high *J*_SC_ and FF observed in PTzBI-*d*F:BTP-TBr devices.

### Thermal properties of acceptors

**Fig. 3:**
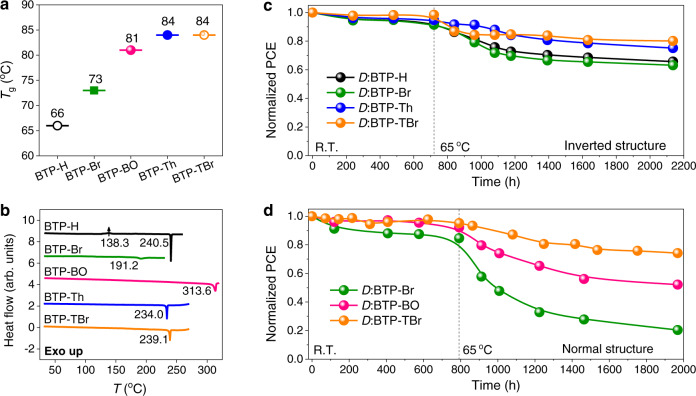
Thermal properties of acceptors and long-term stability of solar cells. **a** *T*_g_ of acceptors as film state extracted from the deviation metric of absorption spectra under different temperatures. **b** DSC thermograms for neat acceptors, obtained from the first heat cycle with a heating rate of 5 K min^−1^. **c** Long-term storage and thermal stability for inverted solar cells. **d** Stability for conventional devices based on acceptors with different side-group hinderance.

To evaluate the thermal tolerance of all five acceptors, glass transition temperature (*T*_g_) was measured in film state by extracting the deviation metric from temperature-dependent absorption spectra (Supplementary Fig. 17). By accumulating the square of optical density difference at each wavelength (see the details in Methods)^42^, the deviation metric for each acceptor film was obtained as a dependence on temperature. The linear fitting of the steep region gives an inflection point (*T*_g_) of 66, 73, and 81 ^o^C for BTP-H, BTP-Br, and BTP-BO, respectively, while BTP-Th, and BTP-TBr deliver a same value of 84 ^o^C (Fig. 3a, Supplementary Fig. 18). The lower *T*_g_ of BTP-H and BTP-Br suggests a higher diffusion coefficient according to the Ade-O’Connor-Ghasemi stability framework^36^. This is further confirmed from grazing incidence wide-angle X-ray scattering (GIWAXS) of neat acceptors. BTP-TBr shows obviously weaker π-π (backbone) packing than that of BTP-Br, revealed by the larger *d*-spacing and lower crystal coherence length (CCL) of (010) peak in out-of-plane (OOP) direction (Supplementary Fig. 19, Supplementary Table 5). This implies a stronger tendency of BTP-Br to diffuse to form aggregates in the blend.

The side-group behavior of twisted acceptor BTP-TBr under thermal stress below *T*_g_ was investigated by temperature-dependent ^1^H NMR in deuterated benzene. When the temperature was elevated from 30 to 72 ^o^C, a gradually changed chemical shift (δ) from 7.23 to 7.20 ppm can be clearly observed for the proton on thienyl group (Supplementary Fig. 20), while the δ of other low-field peaks remain almost unchanged. This suggests the conjugated side group could rotate under heating. Although the side-group behavior in solution is different from that in solid state, it shows us the possible scenario when the solid film experiences higher thermal stress.

Differential scanning calorimetry (DSC) for both neat acceptors and their blends were carried out to discover their thermal behaviors at higher temperatures. To avoid the decomposition of acceptors in DSC traces, the temperature was set below the onset of weight loss determined from thermogravimetric analysis (TGA, Supplementary Fig. 21a). The DSC curves of neat acceptors were depicted in Fig. 3b, where a clear exothermal peak appears at 138.3 ^o^C for BTP-H, indicating the existence of cold crystallization (*T*_c_) before melting (240.5 ^o^C). The other acceptors show only one endothermal peak attributed to the melting point (*T*_m_). Specially, BTP-Br exhibits a significantly low enthalpy of melting (Δ*H*_m_). The low *T*_c_ of BTP-H and the low Δ*H*_m_ for BTP-Br suggest the planar acceptors are not conformationally stable against thermal stress. The DSC results of blends agree well with those for neat acceptors with *T*_m_ slightly shifted (Supplementary Fig. 21b), demonstrating a similar thermal behavior of the acceptors before and after blended with PTzBI-*d*F. Given the DSC of blends were prepared through scraping the dried blend films, the decreased Δ*H*_m_ relative to that of neat acceptors should be due to the interactions between PTzBI-*d*F and acceptors.

### Stability of xylene-processed devices

Long-term stability was evaluated to reveal the influence of side group’s electronic and steric effects on the bulk morphology evolution. Inverted solar cells were employed to reveal the relationship between electronic effect and stability. The set of BTP-H- versus BTP-Br-devices shows a similar trend of efficiency decay, similar to the set of BTP-Th- versus BTP-TBr-devices, with ~66% and ~80% of original efficiency retained, respectively, after a total of ~2200 h aging (Fig. 3c, Supplementary Fig. 22). These results indicate there is only a small influence of electronic effect on the cell stability.

Therefore, we turned to investigate the impact of side-group’s steric effect on stability based on the set of BTP-Br, BTP-BO, and BTP-TBr. Normal structure was used to simultaneously reveal the difference of stability from that in inverted condition. The first ~800 h aging at room temperature degrades the performance by 5% and 9% for BTP-TBr- and BTP-BO-devices, while the degradation for those based on BTP-Br reaches ~15% (Fig. 3d). The subsequent thermal stress at 65 ^o^C for ~1200 h dramatically accelerates the PCE decaying for all devices especially those based on BTP-Br, with only 20% of their original efficiency remained. BTP-BO-devices show a moderate stability with a total decaying of PCE for ~50%, while devices based on BTP-TBr show much better stability with PCE losses of only ~25% during a very long aging period of ~2000 h.

For devices based on the twisted acceptors, especially BTP-TBr, the degraded performance is mainly due to the decay of FF, while the devices based on the planar acceptor (i.e., BTP-Br) show sharp decay of all three parameters during thermal aging (Supplementary Fig. 23). Since BTP-BO showed moderate performance and stability, we only focused on discussing BTP-Br and BTP-TBr to uncover the different degradation mechanisms in devices. The tolerance of BTP-Br- and BTP-TBr-devices under a higher stress of 85 ^o^C was investigated. Still, the *V*_OC_ and FF decay sharply for the former devices within 400 h, while the decaying of those for the latter ones are largely alleviated (Supplementary Fig. 24). The in-situ maximum power point (MPP) tracking was performed for BTP-TBr-devices with normal structure, under one sun illumination generated from a LED light (Supplementary Fig. 25) with the N_2_-filled chamber temperature around 40 ^o^C. After ~250 h photo-aging, the PCE remain 86% of its original value (Supplementary Fig. 26), which is impressive considering the less-robust normal structure, indicating a good tolerance of devices based on twisted acceptors under photo stress and electrical bias.

**Fig. 4:**
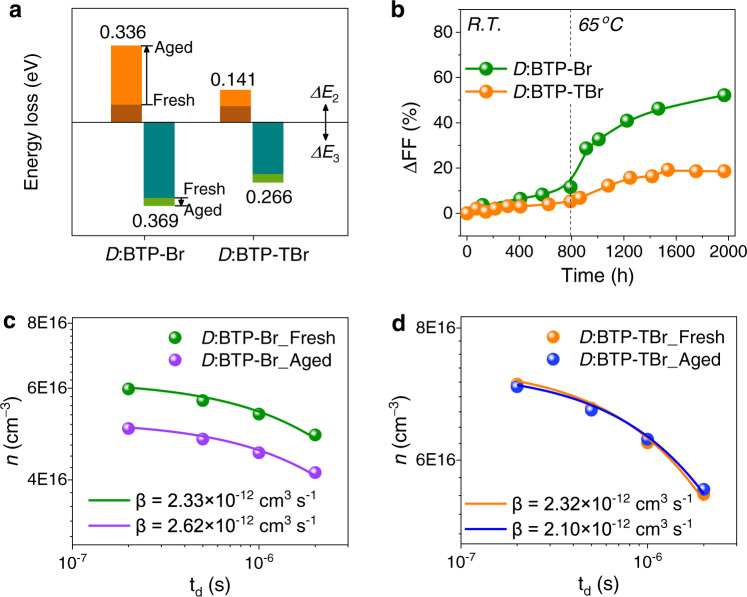
Effect of physical aging on energy loss and charge recombination. **a** Comparison of *V*_OC_ losses for fresh and aged devices. **b** The FF attenuation (ΔFF) against aging time for different devices. Extracted charge carrier density (*n*) as a dependence of delay time (*t*_d_) and 2nd order recombination coefficient (*β*) fitted from *n*-*t*_d_ plots for devices based on BTP-Br (**c**) and BTP-TBr (**d**).

To understand why the *V*_OC_ and FF of BTP-TBr-devices are more robust than those for BTP-Br-counterparts, the EQE and EL quantum yield were measured for aged devices after aging for ~2000 h. The aged devices based on BTP-Br and BTP-TBr show a *V*_OC_ of 0.47 and 0.74 V, respectively (Supplementary Table 6). The EQE fitting generates a slightly enlarged *E*_g_ of 1.431 eV for BTP-Br aged devices. Besides, the emission is largely quenched for BTP-Br-devices with *EQE*_EL_ decreased sharply to 5.80 × 10^−7^, corresponding to a large *ΔE*_3_ of 0.369 eV (Supplementary Fig. 27, Supplementary Table 7 and 8). However, the most obvious loss upon aging is *ΔE*_2_, which damatically increases from 0.077 to 0.336 eV for BTP-Br-devices (Fig. 4a). By comparing the normalized FTPS spectra for devices before and after aging, we attribute the largely increased *ΔE*_2_ to the changed EQE in the sub-bandgap region (i.e., increased *E*_U_), while the spectral shape of FTPS for BTP-TBr-devices is almost unchanged (Supplementary Fig. 28). The increased energetic disorder in planar acceptor-based devices might be due to the formation of some trap states within the bandgap^51^. This should be the result of a substantially changed bulk morphology after long-term aging.

Moreover, delay-time charge extraction by linearly increasing voltage (delaytime-CELIV) was used to investigate the charge recombination in devices by applying varied delay times (*t*_d_) between light pulse and voltage ramp. Through the integration of current overshoot on top of displacement current, the extracted charge carrier density (*n*) based on each *t*_d_ is obtained. Compared with fresh devices, being aged at 85 ^o^C for 7 d shows an obviously decreased *n* at all delay-times for BTP-Br-devices (Fig. 4c), whereas BTP-TBr-ones show only slightly reduced *n* (Fig. 4d). The *n*-*t*_d_ plots for all devices are well fitted by, *n*(*t*_*d*_) = *n*_0_/(1 + *n*_0_*βt*_*d*_), where *β* is the second order recombination coefficient, *n*(*t*) is the charge carrier density at delay-time *t*_*d*_, and *n*_0_ is the initial charge carrier density^52^, The apparently increased *β* for the aged devices based on BTP-Br explains their rapidly declined FF (Fig. 4b). In contrast, no *β* increase can be observed for BTP-TBr-devices, implying a robust morphology for BTP-TBr-blend upon thermal aging.

### Molecular packing and morphological evolution

**Fig. 5:**
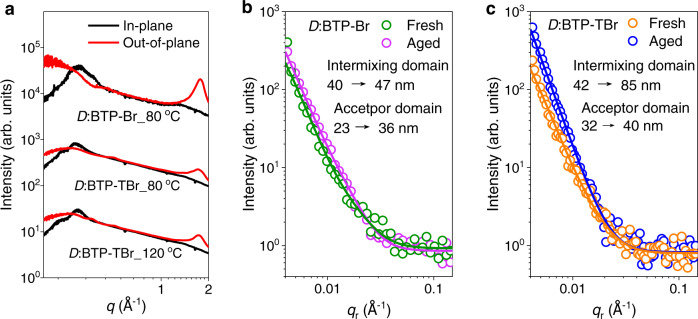
Molecular packing and morphology evolution upon thermal aging. Averaged curves of GIWAXS for blend films based on PTzBI-*d*F (*D*) and different acceptors (**a**). In-plane intensity profiles of GISAXS for fresh and aged blend films based on BTP-Br (**b**) and BTP-TBr (**c**), where solid lines represent the fitting by hard sphere model; the aging induced changes in both intermixing domain size and pure acceptor domain size are shown for clarity.

GIWAXS of blend films was used to correlate molecular packing and crystallization behaviors with their performance and lifetime. The blending with PTzBI-*d*F does not significantly alter the molecular packing of acceptors as in neat condition (Supplementary Fig. 19). When the blends were annealed at 80 ^o^C, a temperature higher than the *T*_g_ of BTP-Br but lower than that of BTP-TBr, the BTP-Br-blend shows a much stronger OOP (010) peak relative to that of BTP-TBr-blend (Supplementary Fig. 29). The fitting of this peak reveals a *d*-spacing of 3.53 Å and a CCL of 34.27 Å for BTP-Br-blend, while BTP-TBr-case shows a larger *d*-spacing of 3.59 Å and a smaller CCL of 22.89 Å (Fig. 5a, Supplementary Table 9).

Considering the peak in high-*q* region of OOP is an overlapping of signals from both donor and acceptor that cannot be deconvoluted, the much better π-π stacking of BTP-Br-blend might be partially derived from the inferior compatibility between PTzBI-*d*F and BTP-Br compared to that between PTzBI-*d*F and BTP-TBr, as revealed by the smaller interfacial energy *γ*_D-A_ (0.15 mN m^−1^) of the latter than that of 0.38 mN m^−1^ for the formers (see contact angle analysis in Supplementary Table 10). When BTP-TBr-blend was annealed at 120 ^o^C, a temperature much higher than the *T*_g_ of BTP-TBr, we observe a slightly increased CCL for π-π stacking with an unchanged *d*-spacing, and meantime a reduced CCL for lamellar stacking. These findings indicate a lower degree of molecular diffusion for BTP-TBr even under high thermal stress, which could be the combined results of large steric hinderance of BTP-TBr and good miscibility between BTP-TBr and polymer donor.

Raman mapping, transmission electron microscopy (TEM), and grazing incidence small-angle X-ray scattering (GISAXS) were used to reveal the impact of steric effect of side group on phase separation and its evolution under aging. Raman spectra show no obvious changes in the characteristic peaks for blends after aging^53,54^, but the mapping of acceptor in blends reveal a homogeneous matrix with less obvious acceptor grains in aged BTP-TBr-blend, whereas slight gathering of acceptor is observed in some local regions for aged BTP-Br-blend (Supplementary Fig. 30–32). The fresh BTP-Br-blend film exhibits a random phase segregation with moderate feature size, which differs a lot from the highly regular texture presented in fresh BTP-TBr-blend (Supplementary Fig. 33). Both BTP-Br- and BTP-TBr-blends show enlarged contrasts after being thermally aged at 85 ^o^C for 7 d, but a more amorphous morphology with most of the original texture disappeared is observed for the latter. The fitting of GISAXS intensity profiles (Supplementary Fig. 34) along the IP direction by a classic model gives both the average acceptor domain size (2R_g-fractal_) and the intermixing domain size (ξ)^55^. The BTP-Br-blend shows an enlarged 2R_g-fractal_ from 23 to 36 nm after thermal aging, but a slightly increased ξ from 40 to 47 nm (Fig. 5b, Supplementary Table S11). The aged BTP-TBr-blend shows a smaller increasement of 2R_g-fractal_ (from 32 to 40 nm) but having a similar value as that for BTP-Br-blend (36 vs. 40 nm). Specially, a dramatically increased ξ (from 42 to 85 nm) is observed for the aged BTP-TBr-blend (Fig. 5c), which is consistent with the Raman and TEM results and might be due to its more sterically hindered conjugated side groups.

## Discussion

Combined with the above results, we summarize the structure–performance–stability framework in the investigated material systems as follows. For devices based on planar acceptors, the components in the mixed phase experienced a slight demixing and purification under thermal stress to form small-scale isolated acceptor domains^56^, due to the high diffusion ability (low *T*_g_) and strong backbone interaction of these planar acceptors as well as relatively low miscibility with polymer donor. The reduced interphase boundaries induced by demixing will decrease the *J*_SC_ of the aged devices because of reduced exciton splitting^57^. Besides, the formation of isolated domains is evidenced by the increased bimolecular recombination coefficient for aged devices based on planar acceptors, which accounts for the decayed FF under thermal aging.

In contrast, devices based on twisted acceptors show obviously larger degree of intermixing in the mixed phase of active layer, because BTP-TBr has relatively higher *T*_g_, weaker backbone stacking, bigger side-group steric hindrance, and better miscibility with polymer donor. As a result, the maintained well-mixed phases do not really affect the charge separation and bimolecular recombination in aged devices, showing better tolerance of *J*_SC_ in the corresponding cells. However, the thermal stress may undermine the charge transport pathways from pure domains, leading to slightly declined fill factors.

In summary, a series of NFAs were developed with varied side groups on outward positions of π-core as an alternative of aliphatic chains. Among those, the pristine and bromo-substituted NFAs show rather planar backbone while the ones possessing conjugated side groups have twisted backbone with large side-group torsion. Owing to the narrow bandgap and low energy loss, the twisted acceptors perform well in xylene-processed solar cells, with the best acceptor delivering a PCE of 18.3%, among the highest PCE reported for non-halogenated solvent processed OPV. Meantime, a decent thermal/photo stability is realized for the acceptors with large structural hinderance. Through the systematical investigation of the relationship between chemical structure/geometry and morphology/performance evolution under external stresses, the importance of side-group steric hinderance of NFAs in acquiring the performance–stability equilibrium of OPV is highlighted.

## Methods

### Materials

All solvents and reagents were used as received from commercial sources without further purification. BTP-BO and PTzBI-*d*F were synthesized according to the methods reported before^34,38^. The synthetic details of dialdehyde intermediates were similar to that reported previously in our group^58^, while the synthetic procedures for the target non-fullerene acceptors were elaborated in the Supplementary Information.

### Fabrication of solar cells

Conventional type solar cells were fabricated with a device structure of indium tin oxide (ITO)/PEDOT:PSS/active layer/PFNDI-Br/silver (Ag), where PEDOT:PSS represents poly(3,4-ethylenedioxythiophene):polystyrene sulfonate and PFNDI-Br is an electron transport layer^59^. Cleaned ITO glasses were treated with oxygen plasma (50 W) for 30 min before coating a layer of PEDOT:PSS (PVP Al 4083). The substrates with ~25 nm PEDOT:PSS film were annealed at 150 ^o^C for 20 min in air and then transferred into N_2_-filled glove box. After cooling, blends of PTzBI-*d*F:acceptor (1:1.2, wt:wt) in chloroform (donor concentration: 5.5 mg mL^−1^) or xylene (7.0 mg mL^−1^) with 0.3 vol% chloronaphthalene (CN) were spin-coated onto the substrates at ~750 rpm and thermal annealed at 100 ^o^C for 10 min, giving an active layer thickness of ~100 nm. Then, a thin layer (~5 nm) of electron collection layer PFNDI-Br was coated atop from its methanol solution (0.5 mg mL^−1^) at 2500 rpm for 20 s. For devices with the inverted structure of ITO/zinc oxide (ZnO)/active layer/molybdenum oxide (MoO_x_)/Ag, ~20 nm ZnO (sol-gel) were spin-coated (4000 rpm) on ITO substrates and annealed at 200 ^o^C for 20 min in air. The deposition of active layers was identical to that described for normal-structure devices, while 6 nm of MoO_x_ was thermal deposited atop as a hole collection layer. Silver electrode with thickness of 100 nm was finally deposited in high vacuum of ~2 × 10^−6^ torr through a shadow mask of 4.14 mm^2^. For accuracy, a non-refractive mask was used to further define the device effective area as 3.30 mm^2^ during the *J* − *V* measurements. *J* − *V* characteristics were measured under a computer controlled Keithley 2400 source meter under 1 sun, AM 1.5 G solar simulator (Enlitech SS-F5). The light intensity was calibrated by a standard silicon solar cell (certified by NREL) to give a value of 100 mW cm^−2^. The EQE spectra were conducted on an Enlitech QE-S EQE system equipped with a standard Si diode, where the monochromatic light was generated from a Newport 300 W lamp source.

### NMR/MS measurement and DFT calculation

Proton nuclear magnetic resonance (^1^H NMR) was recorded on Bruker AVANCE III HD (BBO Probe, 300 MHz) with chloroform-d as solvent. The temperature-dependent 1H NMR was performed at Bruker AVANCE III Nuclear Magnetic Resonance System (400 MHz) with benzene-d6 as solvent. The concentration of the acceptor is ~10 mg mL^–1^, and the temperature ranges from 30–72 ^o^C with an interval of 6 ^o^C. The matrix-assisted laser desorption/ionization time of flight mass spectrometry (MALDI-TOF-MS) was performed on Tempo LC MALDI spotting System (ABI). Density functional theory (DFT) was calculated from the Gaussian 09 package (RB3LYP/6-31 G(d)).

### UV–vis absorption and CV measurements

UV-vis absorption was recorded on a SHIMADZU UV-1700 spectrophotometer equipped with an in-situ sample holder heating system. Cyclic voltammetry was measured on a CHI660A electrochemical workstation equipped with a glass carbon working electrode, a platinum sheet counter electrode, and an Ag/AgCl reference electrode.

### *T*_g_ measurements

Glass transition temperature (*T*_g_) of film samples was obtained by extracting the deviation metric (*DM*) from the temperature-dependent absorption spectra. The *DM* is extracted from the formula, ${{DM}}_{T}=\mathop{\sum }\nolimits_{{\lambda }_{{\min }}}^{{\lambda }_{{\max }}}{({I}_{{RT}}\left(\lambda \right)-{I}_{T}(\lambda ))}^{2}$, where *I*_RT_(λ) and *I*_T_ (λ) represent the absorption intensity of as-cast and annealed films, respectively.

### TGA and DSC measurements

Thermogravimetry analysis (TGA) was recorded on a thermal analyzer (PerkinElmer STA6000) at a heating rate of 5 K min^–1^. Differential scanning calorimetry (DSC) was performed on a Mettler-Toledo-DSC3 analyzer at a heating and cooling rate of 5 and 10 K min^–1^, respectively. The blend sample for DSC was obtained by scratching the corresponding dried blend film from the glass substrate.

### EL and FTPS measurements

EL spectra and quantum efficiency were measured on a REPS Pro system (Enlitech) with a Keithley 2400 external current/voltage source meter connected to support an external electric field. FTPS-EQE was recorded on a FTPS (PECT-600) system (Enlitech), where a low-noise current amplifier was employed to amplify the photocurrent generated from the photovoltaic devices with illumination light modulated by the Fourier transform infrared (FTIR) instrument.

### Delaytime-CELIV measurements

Delay-time charge extraction by linearly increasing voltage (CELIV) was measured on an all-in-one platform of Paios (Fluxim AG). A light pulse with duration of 50 μs generated from an 810 nm LED lamp (light intensity 100%) was applied prior to a voltage ramp of 1 V μs^–1^. The delay time between light pulse and voltage ramp was varied from 0.2 to 10 μs. During the delay time, the open circuit was kept by applying the transient photovoltage signal to ensure no current is flowing.

### Impedance measurements

Capacitance (*C*_p_) was measured on an AC impedance analyzer system (Keysight E4991B) using a capacitor structure of ITO/testing film/Ag at a frequency from 20 Hz to 1 MHz. The relative dielectric constant (*ε*_r_) is calculated according to the formula, *ε*_r_ = *C*_p_*D*/(*Aε*_0_), where *C*_p_ is the measured capacitance, *D* is the film thickness, *A* is the contact area, and *ε*_0_ is the vacuum permittivity.

### Contact angle measurements

Contact angle tests were performed on a Dataphysics OCA40 Micro surface contact angle analyzer using deionized water and diiodomethane as the wetting liquid. Surface energy (γ) was evaluated based on the Owens-Wendt-Rabel-Kaelble (OWRK) methodology. The interfacial energy *γ*_D-A_ is calculated from the formula, ${\gamma }_{{{{{{\rm{D}}}}}}-{{{{{\rm{A}}}}}}}={\gamma }_{{{{{{\rm{D}}}}}}}{{\mbox{+}}}{\gamma }_{{{{{{\rm{A}}}}}}}-\frac{4{\gamma }_{{{{{{\rm{D}}}}}}}^{d}{\gamma }_{{{{{{\rm{A}}}}}}}^{d}}{{\gamma }_{{{{{{\rm{D}}}}}}}^{d}+{\gamma }_{{{{{{\rm{A}}}}}}}^{d}}-\frac{4{\gamma }_{{{{{{\rm{D}}}}}}}^{p}{\gamma }_{{{{{{\rm{A}}}}}}}^{p}}{{\gamma }_{{{{{{\rm{D}}}}}}}^{p}+{\gamma }_{{{{{{\rm{A}}}}}}}^{p}}$, where γ^d^ and γ^p^ represent the surface free energy from the dispersion and polar force, respectively.

### TEM and Raman measurements

TEM was recorded on a transmission electron microscope (FEI/Philips Tecnai 12 BioTWIN) operated at 120 kV. Film sample with a thickness of ~100 nm was loaded on the cooper wire mesh by wetting transfer method. Raman spectra and mapping were recorded n by using a Raman spectrometer (Renishaw inVia Reflex) with a 532 nm DPSS laser.

### GIWAXS and GISAXS measurements

GIWAXS measurements were performed at beamline 7.3.3 at the Advanced Light Source (ALS). The 10-keV X-ray beam was incident at a small grazing angle of 0.11–0.15^o^ to maximize the scattering intensity. The scattered X-rays were detected using a Dectris Pilatus 2 M photon counting detector. GISAXS measurements were carried out with a Xeuss 2.0 SAXS laboratory beamline using a Cu X-ray source (8.05 keV, 1.54 Å) and a Pilatus3R 300 K detector. The samples were prepared on silicon wafer and the incidence angle is 0.2^o^.

### Reporting summary

Further information on research design is available in the Nature Research Reporting Summary linked to this article.

## Supplementary information

Supplementary Information

Reporting Summary

## Data Availability

The relevant data are available from the authors upon request.
