# Supplementary material for: Importance of structural hinderance in performance–stability equilibrium of organic photovoltaics
Source: Nat Commun. 2022 Oct 8;13:5946. doi: 10.1038/s41467-022-33754-3 (PMC9547926; doi:10.1038/s41467-022-33754-3)
Supplement: Supplementary file 1 — Supplementary Information [file 41467_2022_33754_MOESM1_ESM.pdf]

SUPPLEMENTARY INFORMATION

Importance of structural hinderance in performance–stability equilibrium of organic photovoltaics

Fan et al.

SUPPLEMENTARY METHODS

General synthesis method: To a 100 mL round-bottom flask, dialdehyde precursor (0.1 mmol) and 2-(5,6-difluoro-3-oxo-2,3-dihydro-1*H*-inden-1-ylidene)malononitrile (IC-2F, 0.4 mmol) were added under N_2 protection. Then, deoxidized chloroform (30 ml) was added and stirred for a while when pyridine (1 ml) was added. The mixture was kept stirring at 65 °C for 12 h. After removal of chloroform of reaction mixture under reduced pressure, 100 ml methanol was added and the precipitate was collected by filtration. The residue was purified by column chromatography on silica gel using a mixture solvent as eluent (hexane/dichloromethane, vol/vol = 1/1) to give a dark solid.

BTP-H: ^{1}H NMR (300 MHz, $CDCl_3$), δ (ppm): 9.02 (s, 2H), 8.58 (m, 2H), 8.23 (s, 2H), 7.72 (t, J = 5.7 Hz, 2H), 4.78 (d, J = 5.4 Hz, 4H), 2.10 (m, 2H), 0.81-1.26 (m, 92H). MALDI-TOF-MS m/z: $[M+H]^+$ calcd. for $C_{92}H_{107}F_4N_8O_2S_5^+$, 1592.71, found 1593.07.

BTP-Br: ^{1}H NMR (300 MHz, $CDCl_3$), δ (ppm): 9.19 (s, 2H), 8.55 (m, 2H), 7.75 (t, J = 5.4 Hz, 2H), 4.78 (d, J = 5.1 Hz, 4H), 2.17 (m, 2H), 0.81-1.25 (m, 92H).

BTP-Th: ^{1}H NMR (300 MHz, $CDCl_3$), δ (ppm): 9.14 (s, 2H), 8.55 (dd, J_1 = 4.8 Hz, J_2 = 7.5 Hz, 2H), 7.71 (t, J = 5.7 Hz, 2H), 7.38 (s, 2H), 7.36 (s, 2H), 4.77 (d, J = 5.4 Hz, 4H), 2.76 (t, J = 5.4 Hz, 4H), 2.12 (m, 2H), 1.71-1.78 (m, 4H), 0.80-1.44 (m, 118H). MALDI-TOF-MS m/z: $[M+H]^+$ calcd. for $C_{116}H_{143}F_4N_8O_2S_7^+$, 1980.93, found 1981.27.

BTP-TBr: ^{1}H NMR (300 MHz, $CDCl_3$), δ (ppm): 9.09 (s, 2H), 8.55 (dd, J_1 = 4.8 Hz, J_2 = 7.5 Hz, 2H), 7.71 (t, J = 5.7 Hz, 2H), 7.23 (s, 2H), 4.77 (d, J = 5.4 Hz, 4H), 2.71 (t, J = 5.4 Hz, 4H), 2.11 (m, 2H), 1.66-1.74 (m, 4H), 0.80-1.43 (m, 118H). MALDI-TOF-MS m/z: $[M+H]^+$ calcd. for $C_{116}H_{141}Br_2F_4N_8O_2S_7^+$, 2138.75, found 2138.10.

SUPPLEMENTARY FIGURES

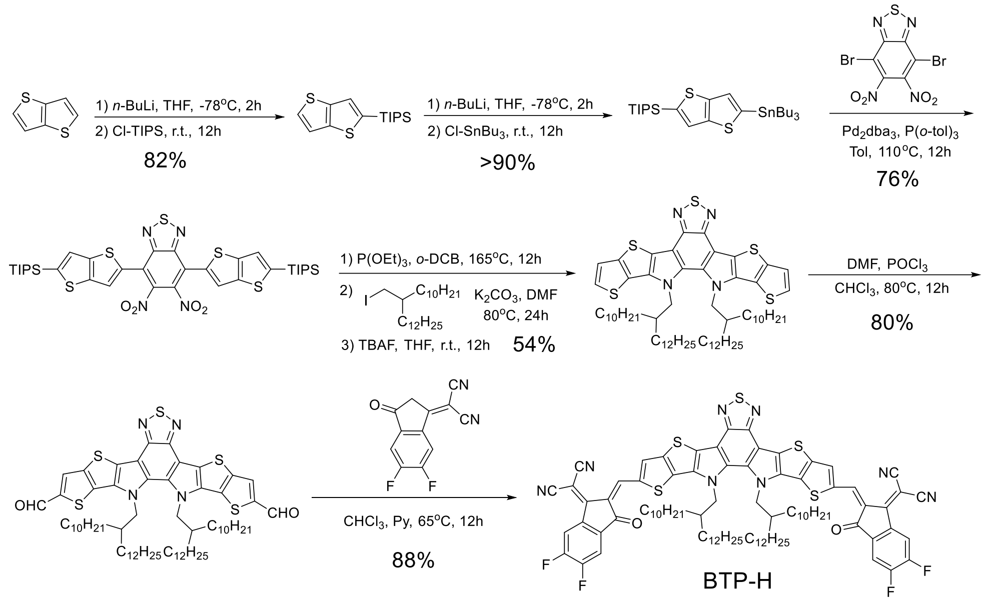

Supplementary Fig. 1 | Synthesis procedure. Synthetic route of BTP-H.

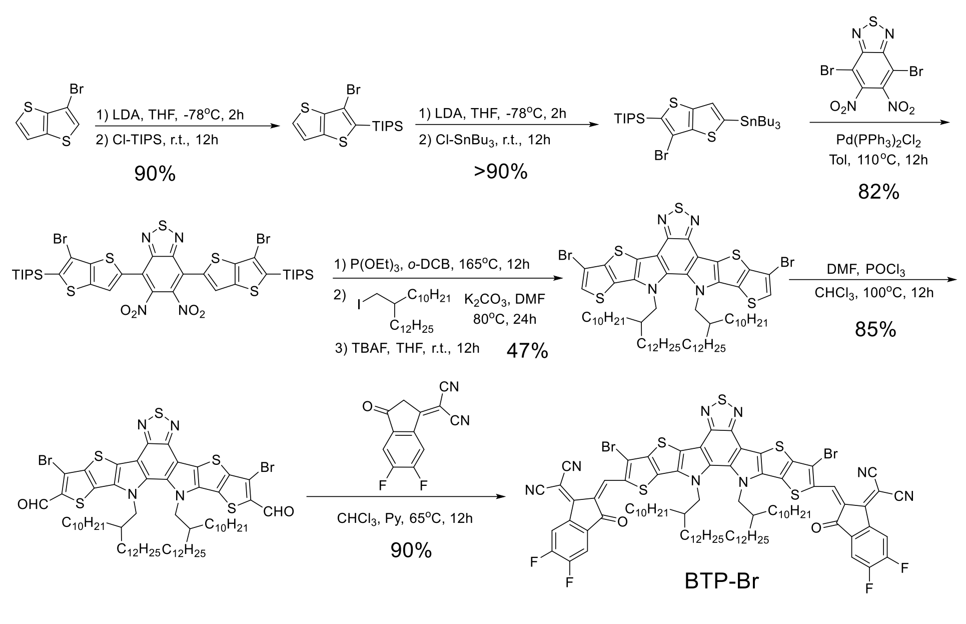

Supplementary Fig. 2 | Synthesis procedure. Synthetic route of BTP-Br.

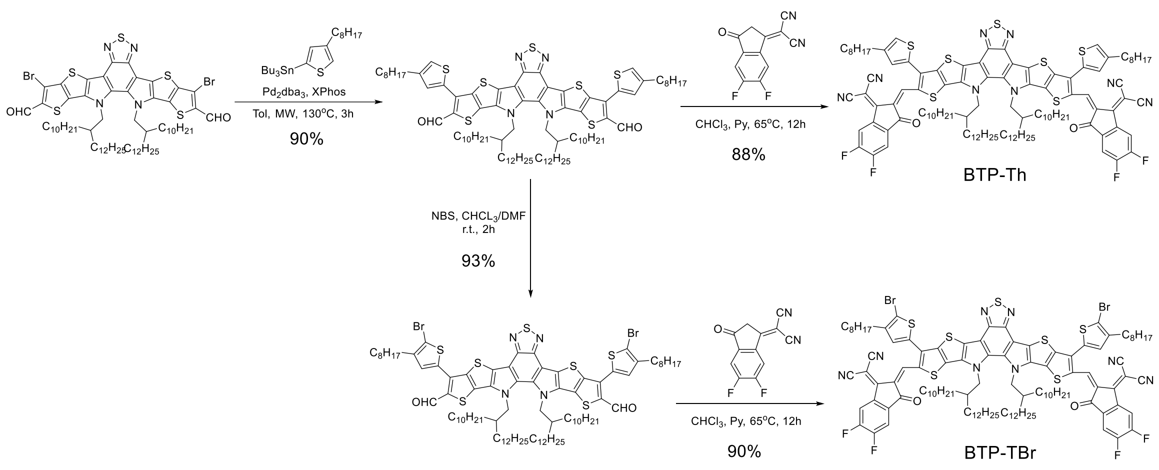

Supplementary Fig. 3 | Synthesis procedure. Synthetic route of BTP-Th and BTP-TBr.

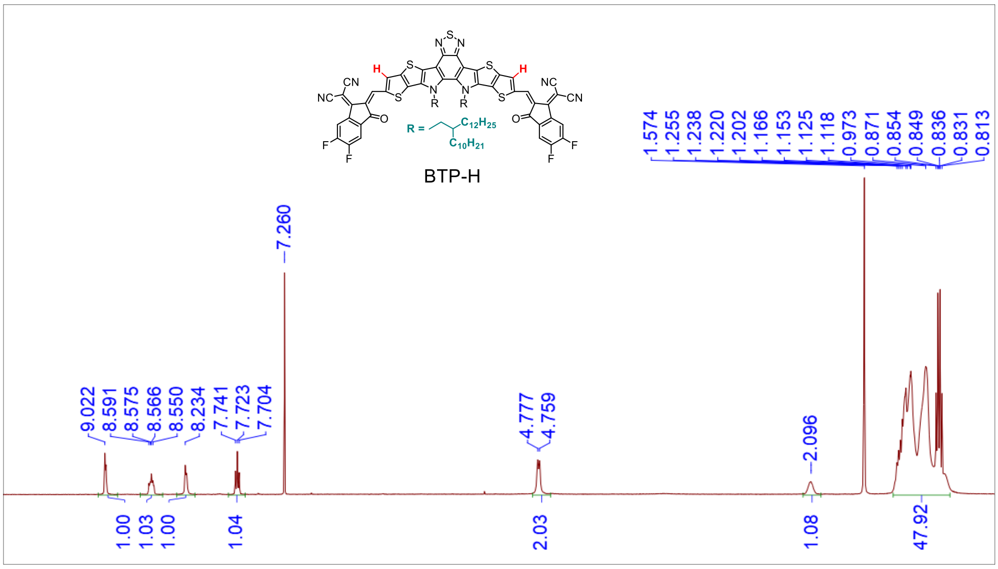

Supplementary Fig. 4 | Structure characterization. ^{1}H NMR spectrum of BTP-H.

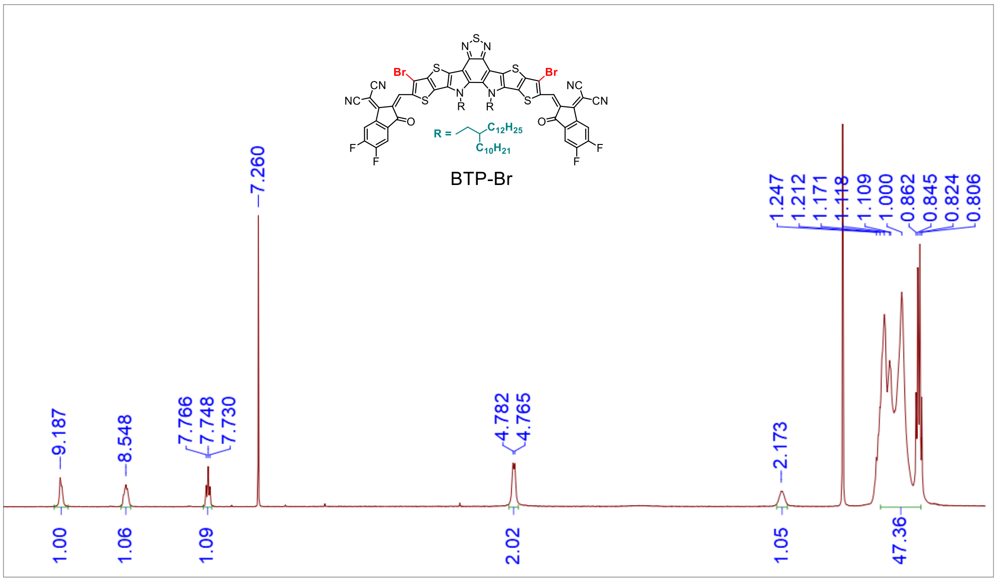

Supplementary Fig. 5 | Structure characterization. ^{1}H NMR spectrum of BTP-Br.

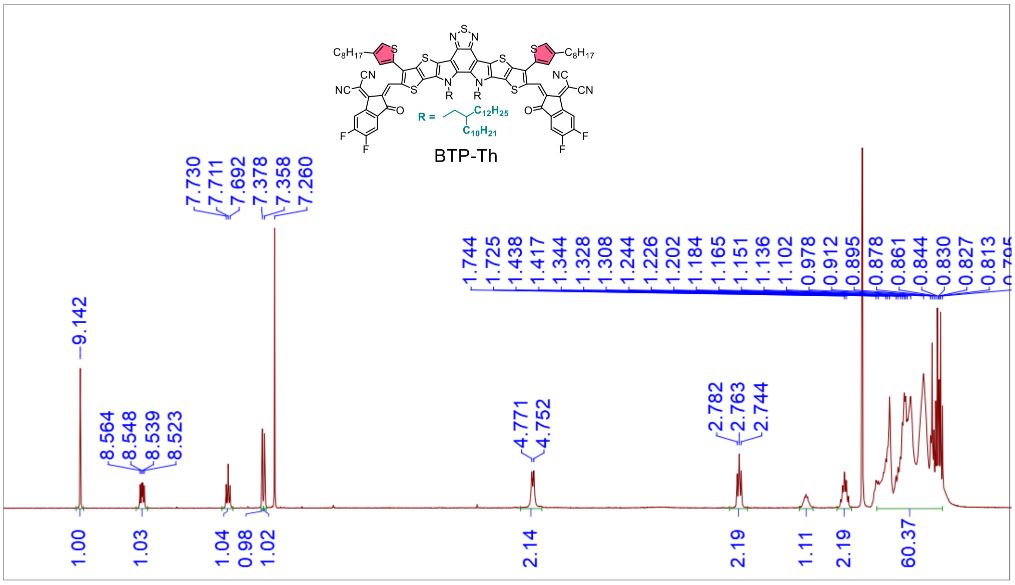

Supplementary Fig. 6 | Structure characterization. ^{1}H NMR spectrum of BTP-Th.

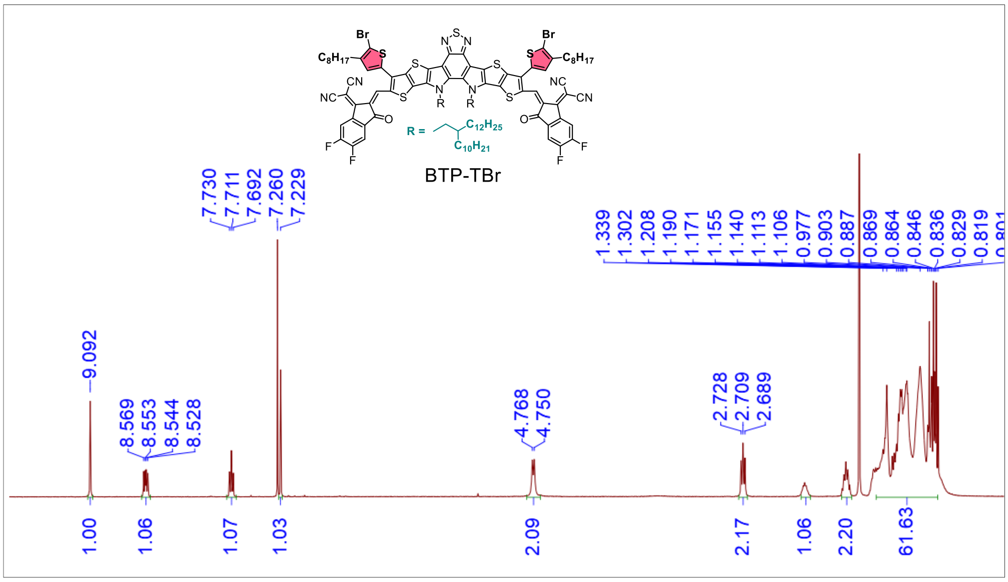

Supplementary Fig. 7 | Structure characterization. ^{1}H NMR spectrum of BTP-TBr.

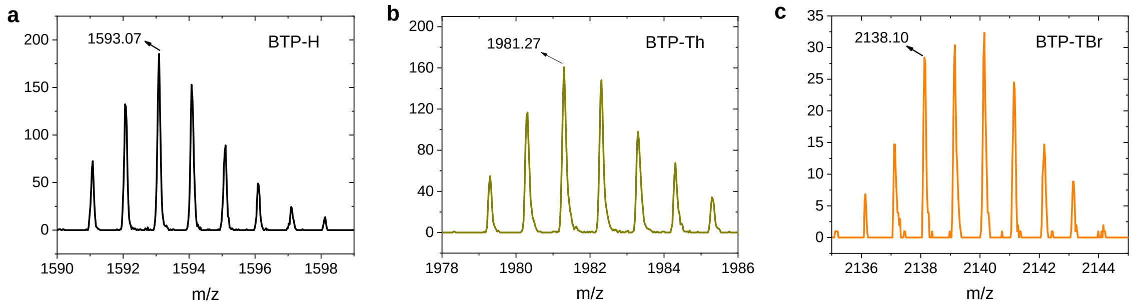

Supplementary Fig. 8 | Structure characterization. MALDI-TOF-MS spectrum of BTP-H (**a**), BTP-Th (**b**), and BTP-TBr (**c**).

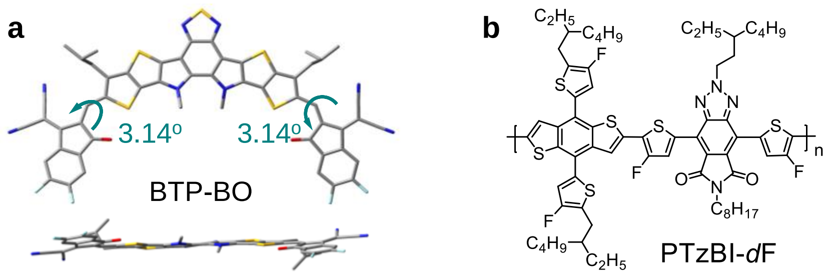

Supplementary Fig. 9 | Chemical structure and geometry. (**a**) Molecular conformation of BTP-BO calculated from the Gaussian 09 package (RB3LYP/6-31G(d)). (**b**) Structure of polymer donor used to match with the acceptors.

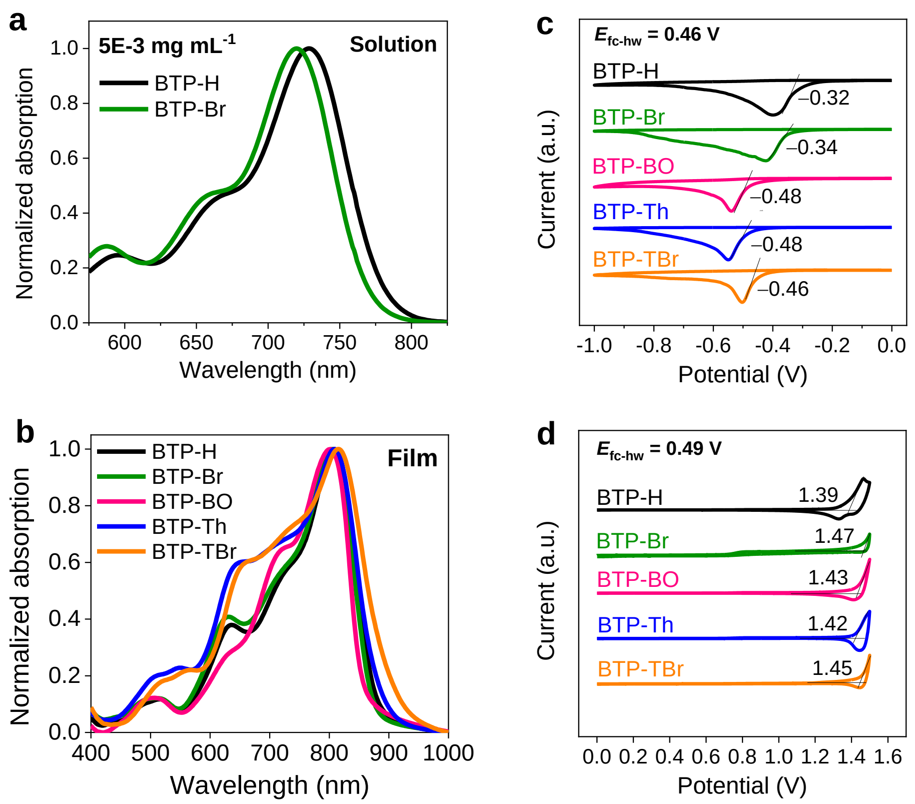

Supplementary Fig. 10 | Photoelectric properties of various acceptors. Normalized absorption for various acceptors in chloroform solution (**a**) and as solid film (**b**). LUMO (**c**) and HOMO (**d**) energy levels determined from CV.

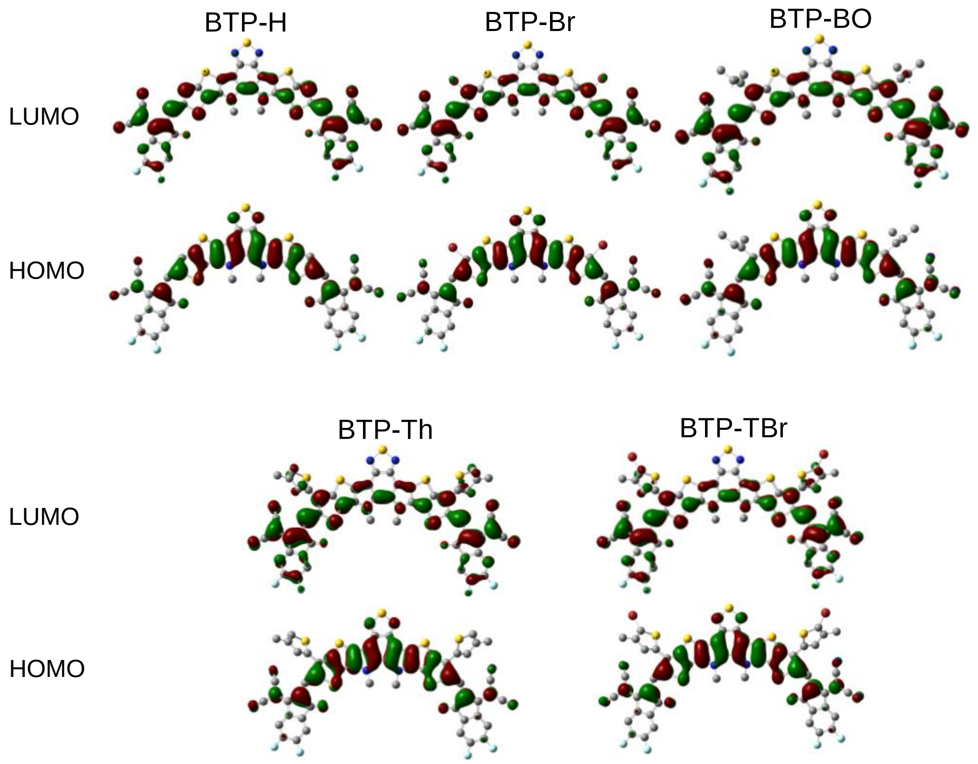

Supplementary Fig. 11 | DFT results of various acceptors. Molecular frontier orbital distributions of various acceptors calculated from the Gaussian 09 package (RB3LYP/6-31G(d)).

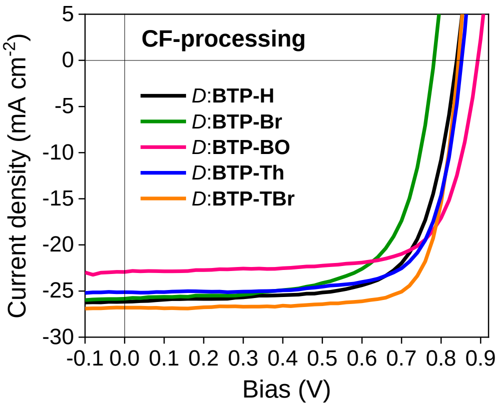

Supplementary Fig. 12 | Photovoltaic performance for chloroform-processed devices. *J*–*V* curves for conventional solar cell devices based on various acceptors.

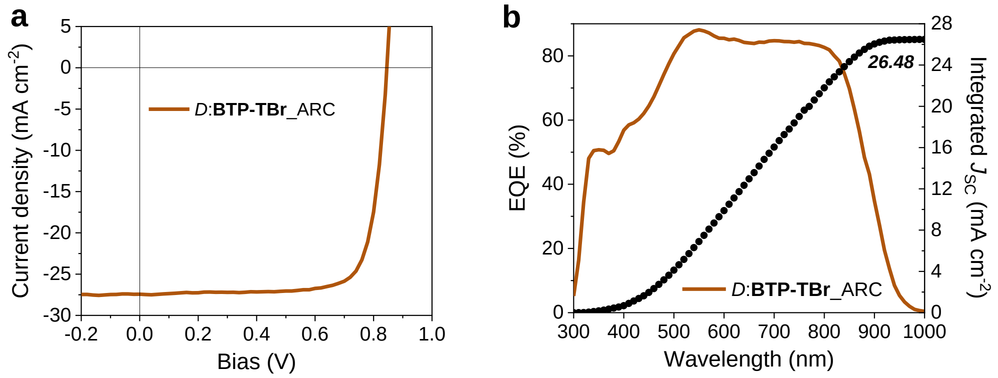

Supplementary Fig. 13 | Photovoltaic performance for xylene-processed devices with MgF_2 ARC. *J*–*V* curve (**a**) and EQE (**b**) for conventional solar cells based on BTP-TBr.

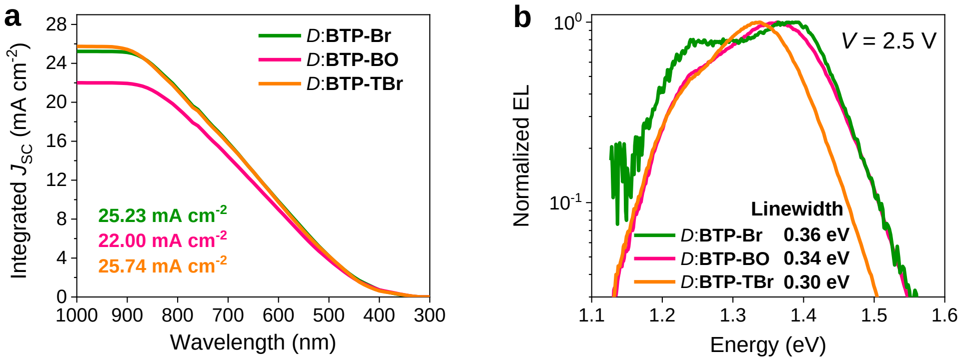

Supplementary Fig. 14 | EQE and EL for xylene-processed devices. (**a**) Integrated J_{SC} from EQE for devices based on various acceptors. (**b**) Emission width determined from the difference between the photon energies where the normalized EL reaches 1×10^{-1}.

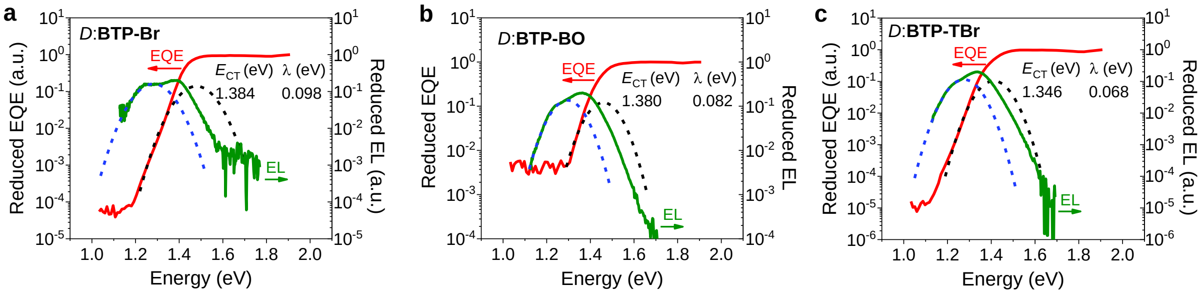

Supplementary Fig. 15 | Analysis of CT-state energy (E_{CT}). E_{CT} for devices based on BTP-Br (**a**), BTP-BO (**b**), and BTP-TBr (**c**), determined from Gaussian fitting from CT emission and absorption spectra according to Marcus theory.

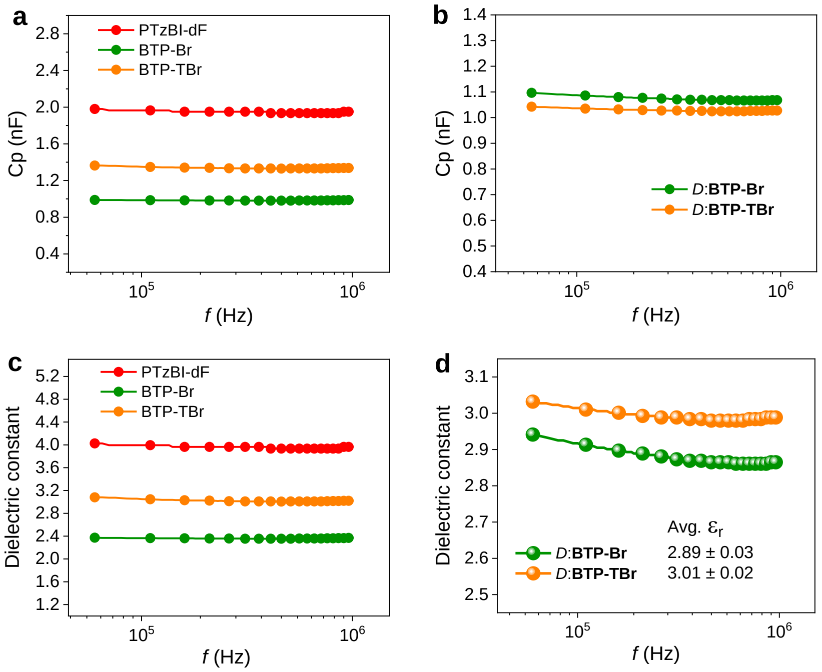

Supplementary Fig. 16 | Impedance results and dielectric constants. Capacitance–frequency plots for devices based on neat donor and acceptor (**a**) and their blends (**b**). The film thickness for PTzBI-*d*F, BTP-Br, BTP-TBr, *D*:BTP-Br, and *D*:BTP-TBr is 72, 85, 80, 95, and 103 nm, respectively. Dielectric constant as a dependence on frequency for neat donor and acceptor (**c**) and their blends (**d**).

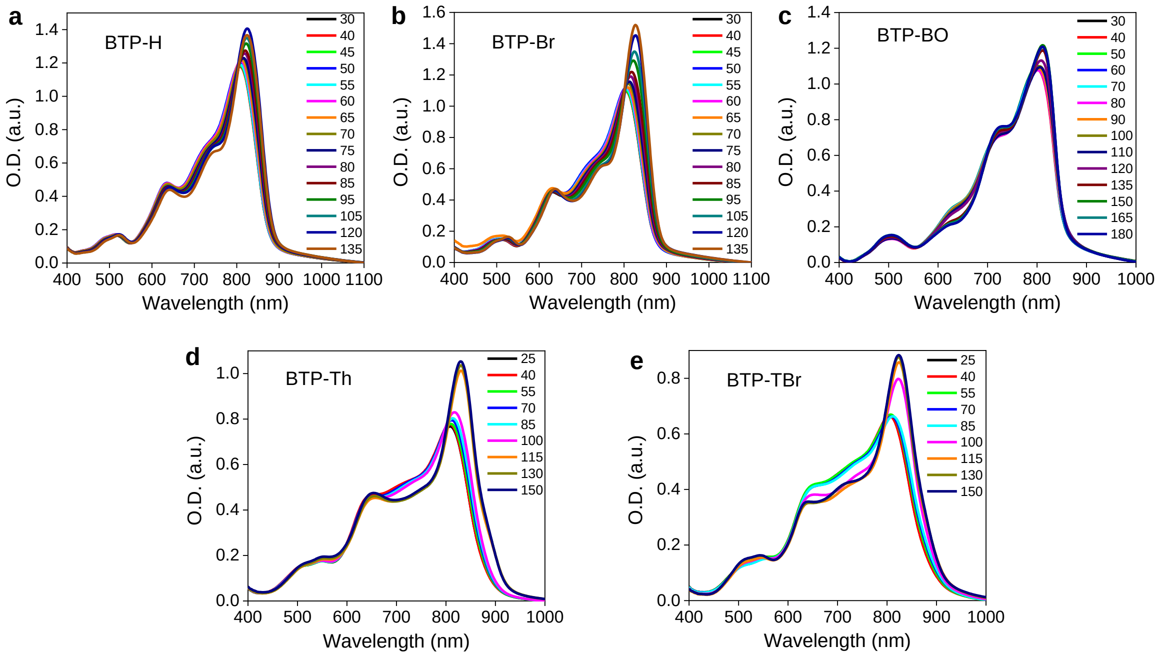

Supplementary Fig. 17 | Temperature-dependent absorption spectra. Film absorption measured under different temperatures for BTP-H (**a**), BTP-Br (**b**), BTP-BO (**c**), BTP-Th (**d**), BTP-TBr (**e**).

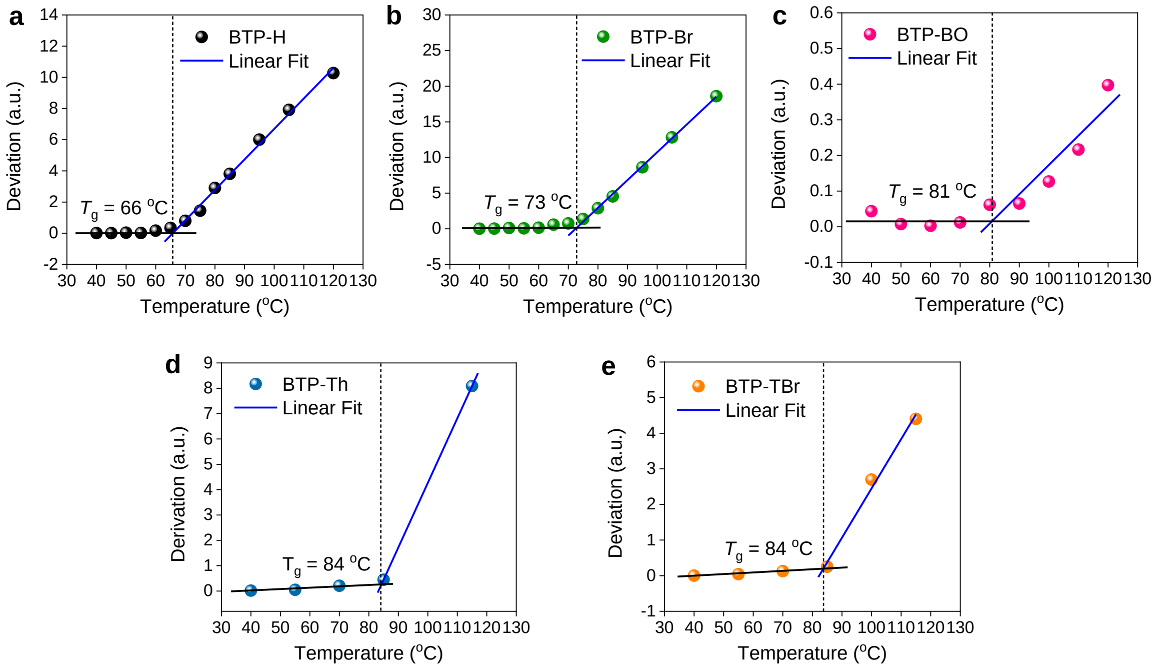

Supplementary Fig. 18 | Determination of T_g for acceptor films. Deviation metric of absorption spectra under different temperatures for BTP-H (**a**), BTP-Br (**b**), BTP-BO (**c**), BTP-Th (**d**), BTP-TBr (**e**).

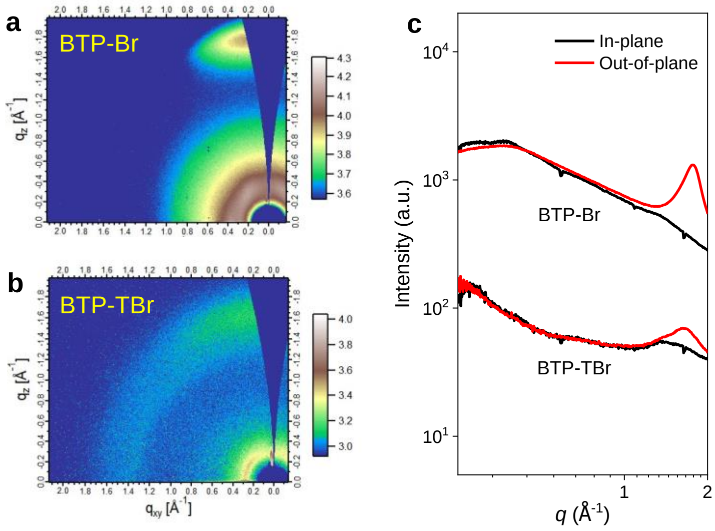

Supplementary Fig. 19 | Molecular packing of neat acceptors. 2D GIWAXS patterns for BTP-Br (**a**) and BTP-TBr (**b**). Relevant average curves for neat acceptor films (**c**).

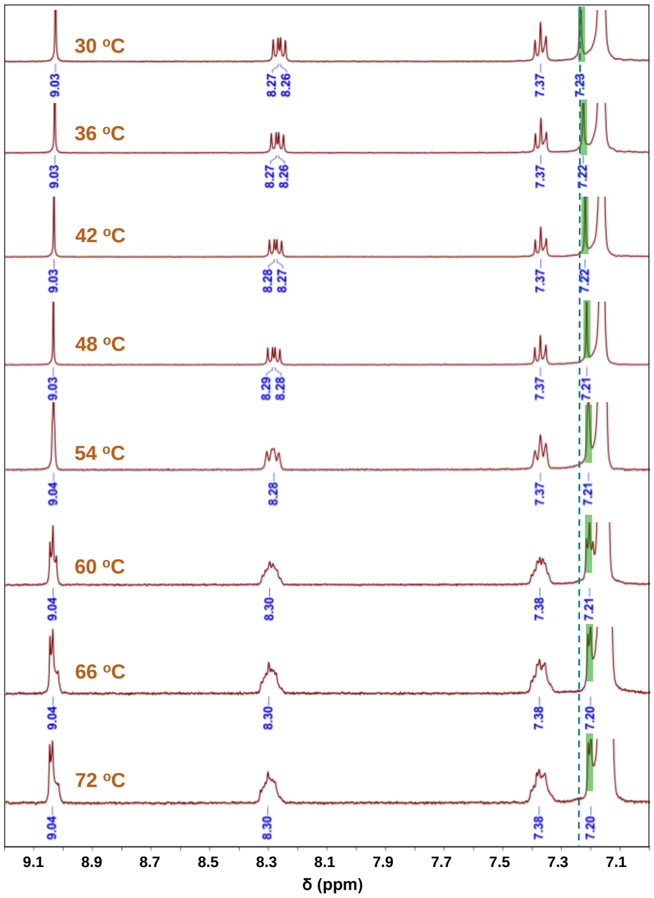

Supplementary Fig. 20 | Change of ^{1}H NMR spectra under different temperatures. Temperature-dependent ^{1}H NMR for BTP-TBr in benzene-d6, where the intense peak at 7.16 ppm belongs to the proton of the solvent.

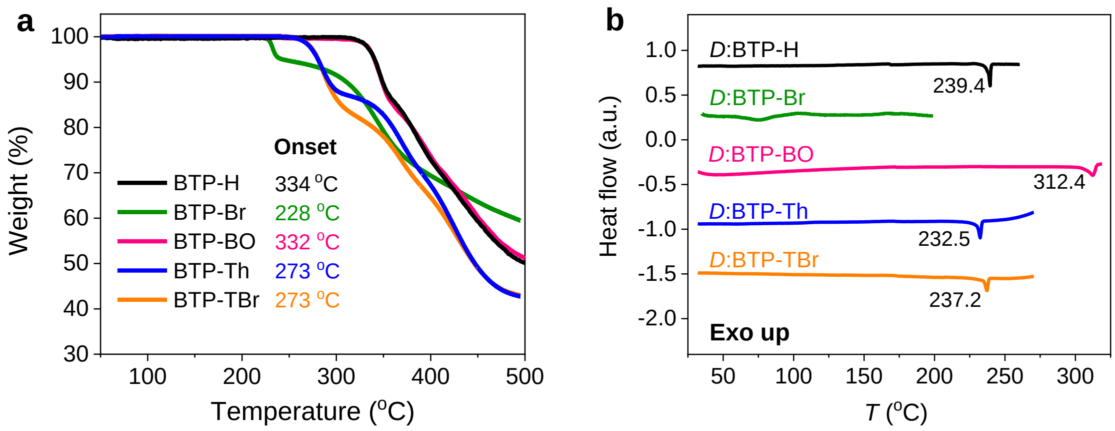

Supplementary Fig. 21 | Thermal properties of acceptors and their blends. Thermogravimetric analysis (TGA) for various acceptors (**a**) and differential scanning calorimetry (DSC) for blends based on PTzBI-*d*F (*D*) and various acceptors. (**b**).

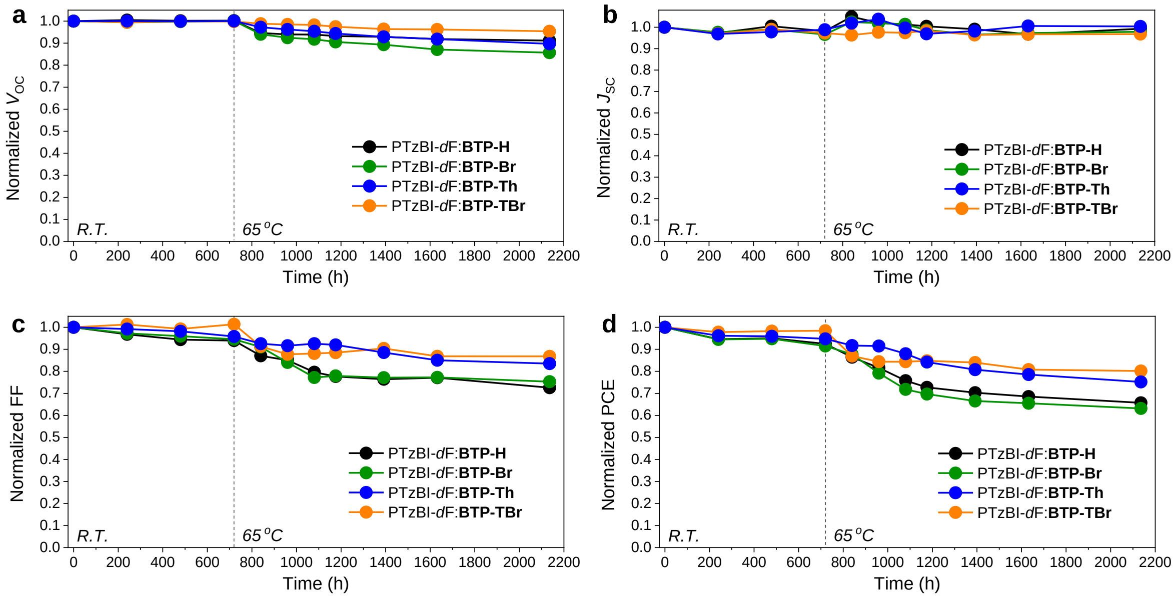

Supplementary Fig. 22 | Device stability under long-term aging. The evolution of V_{OC} (**a**), J_{SC} (**b**), FF (**c**), and PCE (**d**) for inverted-structure solar cells aged at 65 °C.

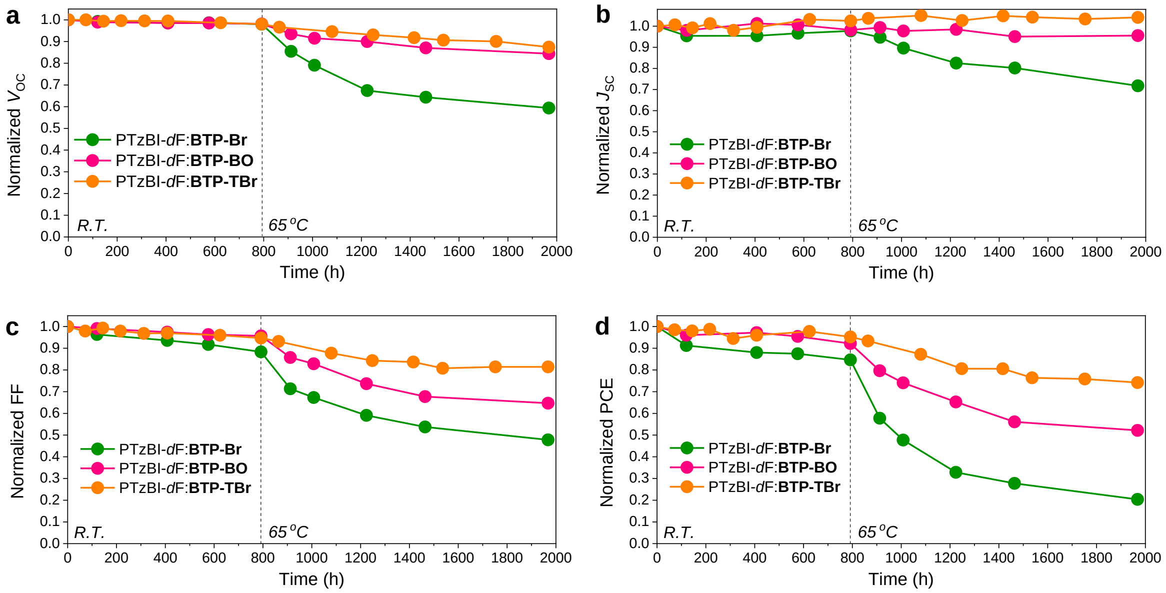

Supplementary Fig. 23 | Device stability under long-term aging. The evolution of V_{OC} (**a**), J_{SC} (**b**), FF (**c**), and PCE (**d**) for normal-structure solar cells aged at 65 °C.

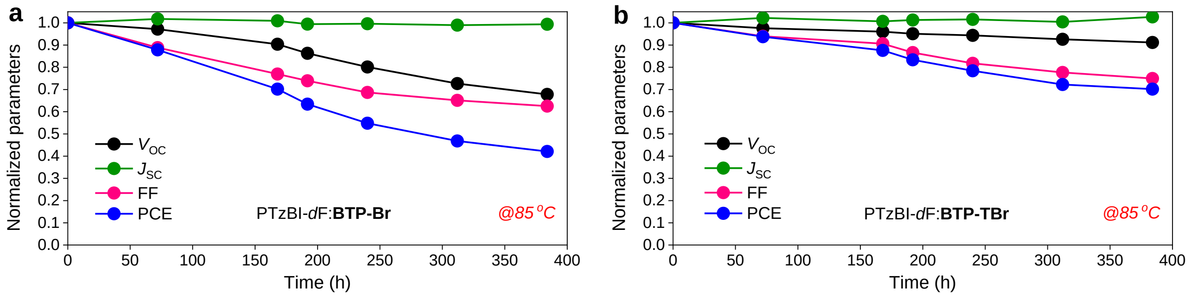

Supplementary Fig. 24 | Device stability under long-term aging. Photovoltaic parameters evolution at 85 °C for normal-structure solar cells based on PTzBI-*d*F:BTP-Br (**a**) and PTzBI-*d*F:BTP-TBr (**b**).

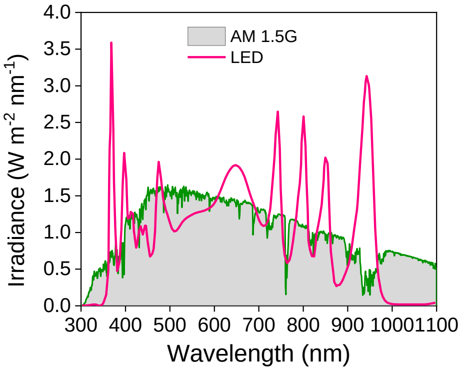

Supplementary Fig. 25 | Light source used for photostability measurement. The spectrum for the LED light used for the MPP tracking, as a comparison to the AM 1.5G irradiance.

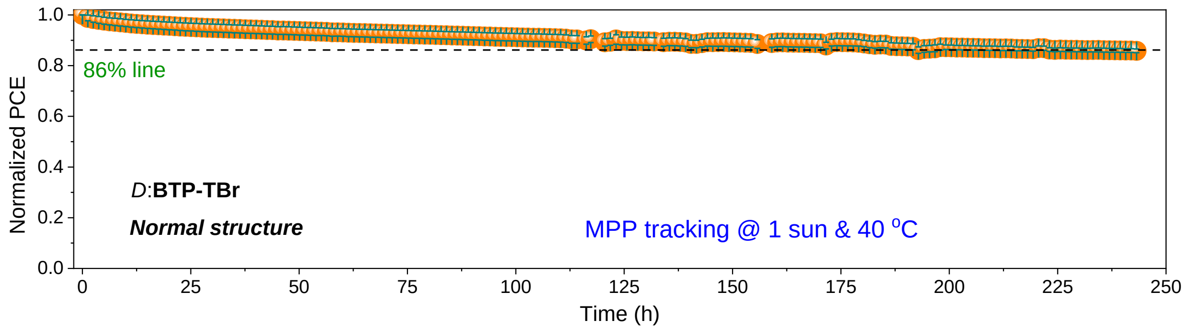

Supplementary Fig. 26 | Photostability under 1 sun illumination. MPP tracking for normal-structure devices based on BTP-TBr under 1 sun illumination at ~40 ºC; the error bar is standard deviation for the statistics with n value of 4.

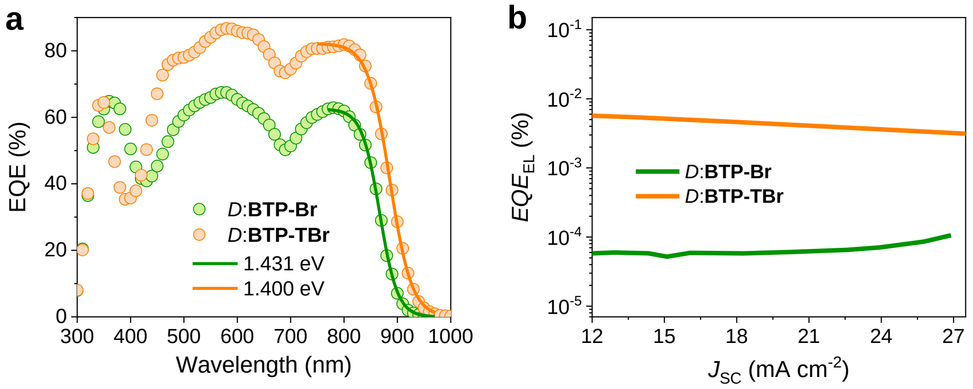

Supplementary Fig. 27 | EL and EQE spectra for aged devices. EL quantum efficiency (**a**) and EQE (**b**) for xylene-processed devices after consecutively shelf and thermal aging for ~2000 h.

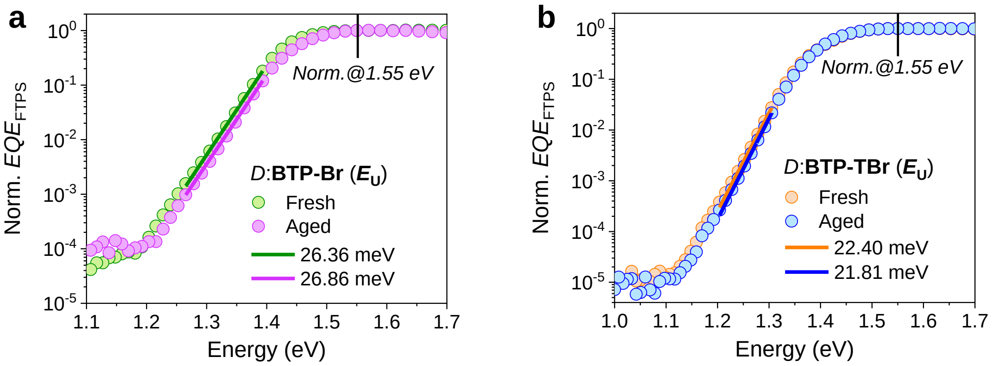

Supplementary Fig. 28 | FTPS spectra for fresh and aged devices. FTPS for xylene-processed devices based on BTP-Br (**a**) and BTP-TBr (**b**) before and after consecutively shelf and thermal aging for ~2000 h.

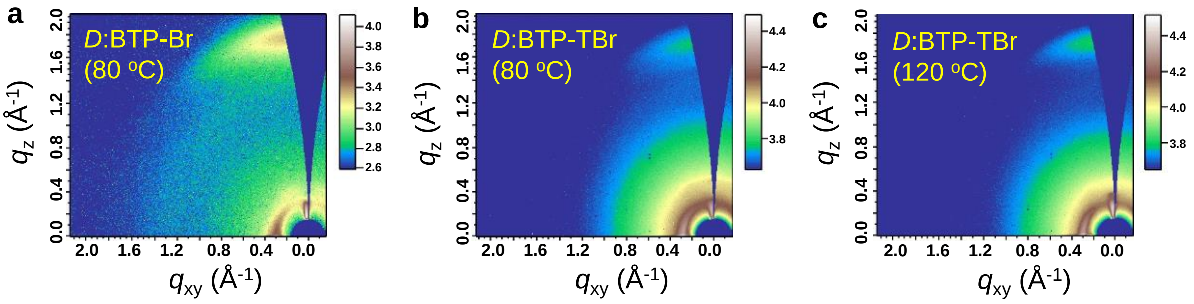

Supplementary Fig. 29 | Molecular packing in blend films under varied thermal stress. Two-dimensional GIWAXS patterns for blend films based on BTP-Br (**a**) and BTP-TBr (**b**, **c**).

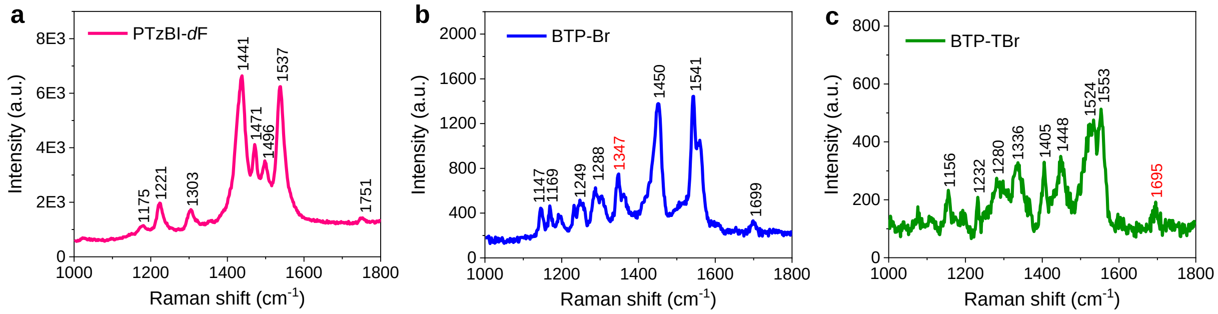

Supplementary Fig. 30 | Raman spectra for PTzBI-*d*F (**a**), BTP-Br (**b**), and BTP-TBr (**c**) neat films.

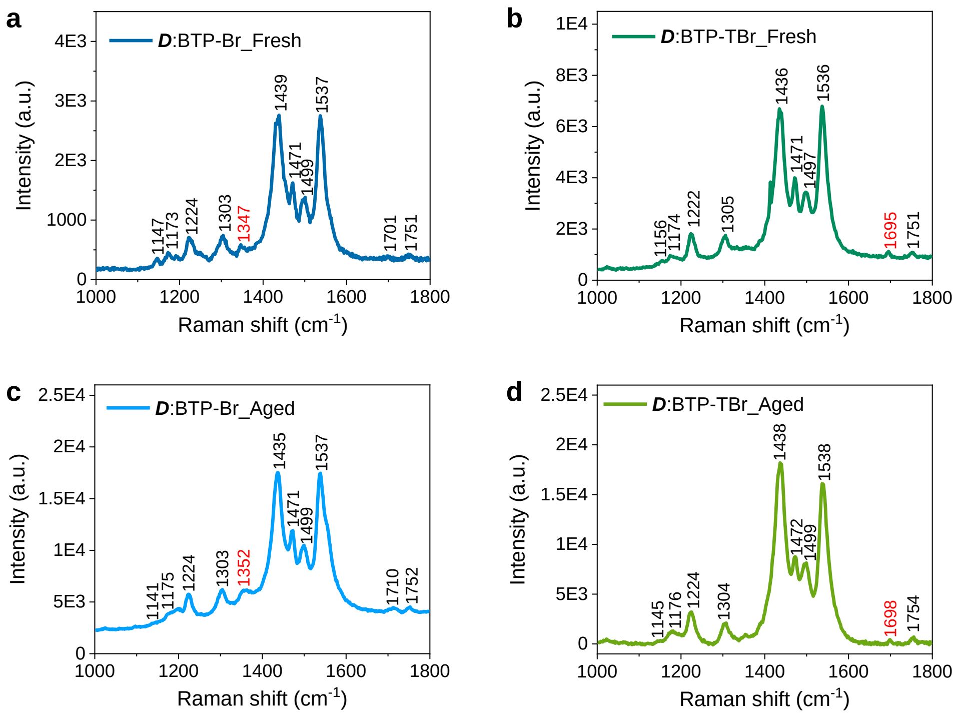

Supplementary Fig. 31 | Raman spectra for blend films of PTzBI-*d*F:BTP-Br (**a**, **c**) and PTzBI-*d*F:BTP-TBr (**b**, **d**) before (**a**, **b**) and after (**c**, **d**) thermal aging at 85 °C for 7 d.

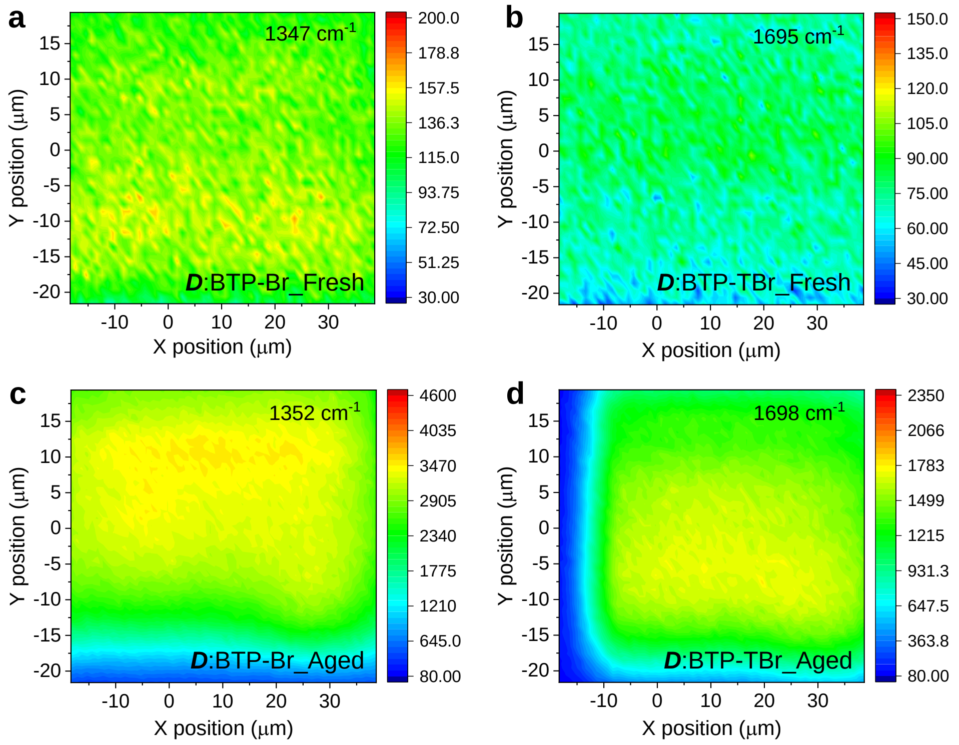

Supplementary Fig. 32 | Raman mapping (60 × 40 μm^2). Distribution of BTP-Br (**a**, **c**, detected from characteristic peak at ~1347 cm^{-1}) and BTP-TBr (**b**, **d**, detected from characteristic peak at ~1695 cm^{-1}) in blend films before (**a**, **b**) and after (**c**, **d**) aging at 85 °C for 7 d. Color bar represents Raman intensity.

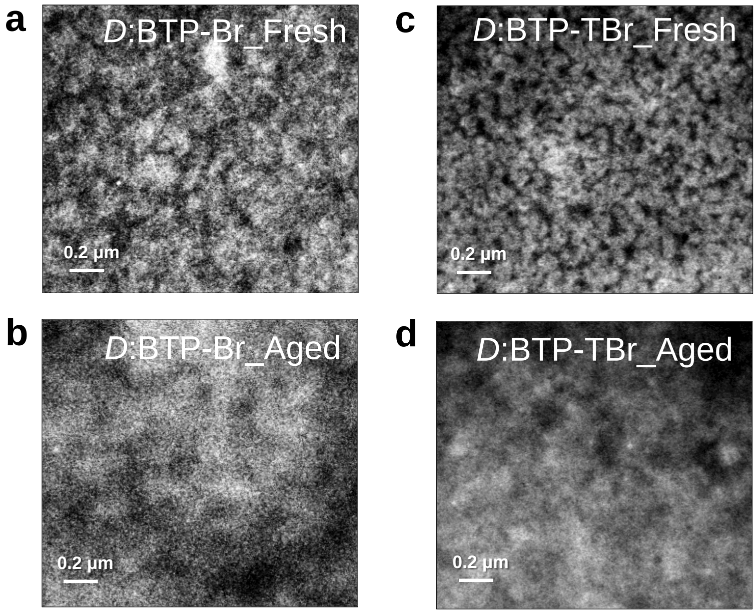

Supplementary Fig. 33 | Phase-separated morphology for fresh and aged films. TEM images for fresh (**a**, **c**) and aged (**b**, **d**) blend films based on BTP-Br (**a**, **b**) and BTP-TBr (**c**, **d**), where aged films are annealed at 85 ^{o}C for 7 d.

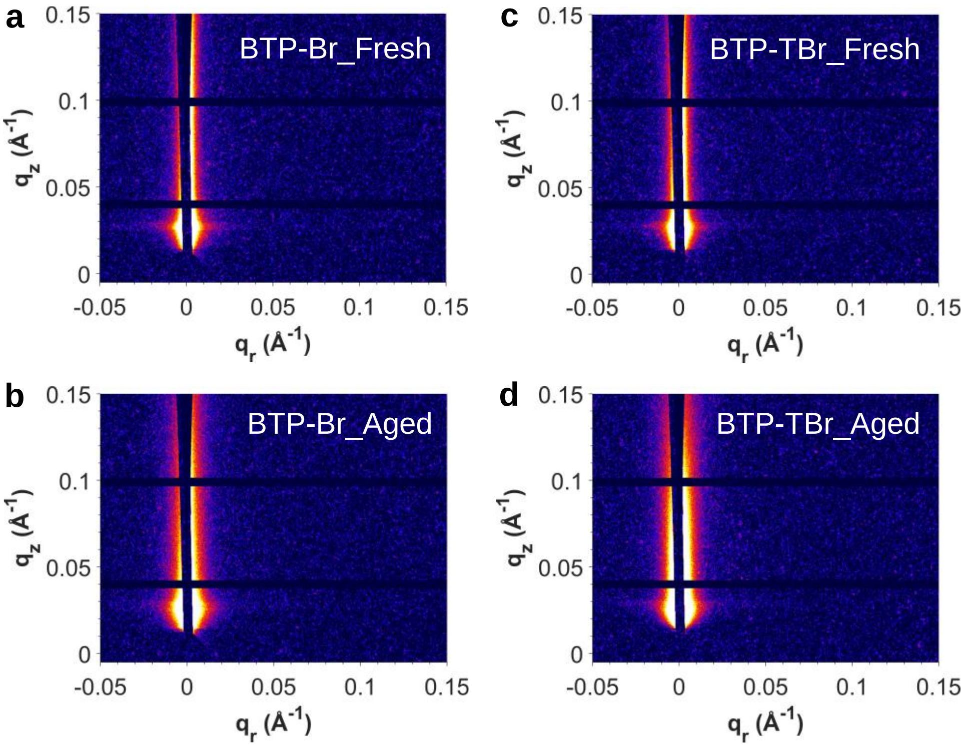

Supplementary Fig. 34 | 2D GISAXS patterns. GISAXS for fresh (**a**, **c**) and aged (**b**, **d**) blend films based on PTzBI-*d*F:BTP-Br (**a**, **b**) and PTzBI-*d*F:BTP-TBr (**c**, **d**), where aged films are annealed at 85 °C for 7 d.

SUPPLEMENTARY TABLES

Supplementary Table 1 | Parameters obtained from density functional theory (DFT), cyclic voltammetry (CV), and temperature-dependent absorption.

Acceptor	Backbone dihedral angle	Lateral-group torsion angle	LUMO[a] (eV)	HOMO[a] (eV)	LUMO[b] (eV)	HOMO[b] (eV)	E_g[c] (eV)
BTP-H	0.20°	/	−4.02	−5.70	−3.62	−5.67	1.434
BTP-Br	0.18°	/	−4.00	−5.78	−3.69	−5.76	1.441
BTP-BO	3.14°	/	−3.86	−5.74	−3.55	−5.60	1.456
BTP-Th	10.68°	43.68°	−3.86	−5.73	−3.52	−5.57	1.429
BTP-TBr	10.23°	43.27°	−3.88	−5.76	−3.60	−5.64	1.401

[a]Energy levels obtained from CV. [b]Energy levels calculated from DFT. [c]Optical bandgap determined from Tauc plots.

Supplementary Table 2 | Photovoltaic parameters for chloroform-processed devices based on PTzBI-*d*F (*D*) and different acceptors.

Blend	V_{OC} (V)[a]	J_{SC} (mA cm^{-2})[a]	FF (%)[a]	PCE (%)[a]
D:BTP-H	0.840 (0.837±0.004)	26.2 (25.9±0.6)	70.5 (70.0±1.4)	15.5 (15.2±0.3)
D:BTP-Br	0.782 (0.784±0.002)	25.8 (25.2±0.3)	67.6 (67.9±1.2)	13.7 (13.4±0.3)
D:BTP-BO	0.893 (0.894±0.003)	22.9 (22.6±0.3)	72.7 (72.4±1.0)	14.9 (14.7±0.3)
D:BTP-Th	0.852 (0.856±0.007)	25.1 (25.0±0.2)	73.7 (72.6±0.8)	15.8 (15.5±0.2)
D:BTP-TBr	0.844 (0.844±0.004)	26.8 (26.5±0.3)	77.7 (77.2±0.6)	17.6 (17.3±0.2)

[a]The value in bracket is average of at least 8 individual devices; the error is defined as standard deviation.

Supplementary Table 3 | The integrated J_{SC} from EQE and the parameters extracted from the sigmoidal fitting of EQE spectra in the low photon energy region.

Device	A_m^a	λ_g^a (nm)	λ_s^a (nm)	E_g^b (eV)
D:BTP-Br	80.90	873.4	40.33	1.420
D:BTP-BO	73.94	853.5	57.54	1.453
D:BTP-TBr	80.98	887.5	51.13	1.397

[a]The parameters are fitted from the sigmoid function, $EQE\ (\lambda) = A_m/(1 + \exp(2.63(\lambda - \lambda_g)/\lambda_s))$, where λ_g is the bandgap wavelength, A_m is related with the maximum EQE, and λ_s expresses the broadening of the absorption threshold in the EQE spectrum. [b]The bandgap of blends is calculated from, $E_g = 1240/\lambda_g$.

Supplementary Table 4 | The parameters extracted from the exponential fitting of FTPS-EQE spectra in the low photon energy region.

Device	α_0^a	E_g^a (eV)	E_U^a (meV)
D:BTP-Br	0.015	1.215	27.62±0.22
D:BTP-BO	0.439	1.309	27.08±0.70
D:BTP-TBr	0.007	1.179	22.84±0.28

[a]The parameters are fitted from the Urbach rule, $\alpha(E) = \alpha_0\exp((E - E_g)/E_U)$, where α_0 is absorption coefficient at band edge, E_g is the calculated bandgap, and E_U is the Urbach energy.

Supplementary Table 5 | Molecular packing parameters for neat films derived from GIWAXS fitting.

Acceptor	Direction	Location ($Å^{-1}$)	*d*-spacing (Å)	FWHM ($Å^{-1}$)	CCL[a] (Å)
BTP-Br	OOP (010)	1.78	3.53	0.185	30.57
BTP-TBr	IP (100)	0.28	22.44	0.066	85.68
	OOP (010)	1.63	3.85	0.196	28.85

[a]Crystal coherence length (CCL) was calculated according to Scherrer equation CCL = $2\pi K\Delta q$, where K is the shape factor ($K = 0.9$) and Δq is the full width at half-maximum (FWHM) of diffraction peak.

Supplementary Table 6 | Photovoltaic parameters for xylene-processed devices after consecutive shelf and thermal aging for ~2000 h.

Blend	V_{OC} (V)	J_{SC} (mA cm^{-2})	FF (%)	PCE (%)
D:BTP-Br	0.470	18.19	33.45	2.86
D:BTP-TBr	0.739	27.15	62.64	12.57

Supplementary Table 7 | Parameters extracted from the sigmoidal fitting of EQE spectra for xylene-processed devices after consecutive shelf and thermal aging for ~2000 h.

Blend	A_m	λ_g (nm)	λ_s (nm)	E_g (eV)
D:BTP-Br	62.48	866.8	43.84	1.431
D:BTP-TBr	82.21	885.8	53.82	1.400

Supplementary Table 8 | V_{OC} and energy losses for xylene-processed solar cells based on PTzBI-*d*F donor after consecutive shelf and thermal aging for ~2000 h.

Acceptor	E_g (eV)	EQE_{EL} @J_{SC}	$\Delta V_{OC,\ nr}$ (V)	$V_{OC,\ SQ}$ (V)	$V_{OC,\ meas}$ (V)	$V_{OC,\ rad}$ (V)	ΔE_1 (eV)	ΔE_2 (eV)	ΔE_3 (eV)	ΔE_{tot} (eV)
BTP-Br	1.431	5.80×10^{-7}	0.369	1.175	0.470	0.839	0.256	0.336	0.369	0.961
BTP-TBr	1.400	3.20×10^{-5}	0.266	1.146	0.739	1.005	0.254	0.141	0.266	0.661

Supplementary Table 9 | Molecular packing parameters for blend films derived from GIWAXS fitting.

Blend	Thermal annealing	Direction	Location ($Å^{-1}$)	*d*-spacing (Å)	FWHM ($Å^{-1}$)	CCL[a] (Å)
D:BTP-Br	80 °C	IP (100)	0.29	21.67	0.068	83.16
		OOP (010)	1.78	3.53	0.165	34.27
D:BTP-TBr	80 °C	IP (100)	0.29	21.67	0.068	83.16
		OOP (010)	1.75	3.59	0.247	22.89
D:BTP-TBr	120 °C	IP (100)	0.30	20.94	0.076	74.41
		OOP (010)	1.75	3.59	0.190	29.76

Supplementary Table 10 | Surface energy of materials calculated from contact angle analysis.

Film	Contact angle (H_2O) [°]	Contact angle (CH_2I_2) [°]	γ^d [mN/m]	γ^p [mN/m]	γ [mN/m]	γ_{D-A} [mN/m]
PTzBI-*d*F	103.8/105.6/105.9	48.7/51.9/50.2	36.73	0.76	37.49	–
BTP-Br	99.9/99.0/99.8	48.6/47.2/46.0	37.52	0.17	37.69	0.38
BTP-TBr	102.4/101.3/101.1	48.6/46.6/48.4	37.57	0.36	37.93	0.15

Supplementary Table 11 | Domain sizes for blend films before and after aged at 85 °C for 7d, obtained from the fitting of the GISAXS intensity profiles.

Blend film	Aging condition	Acceptor domain size $2R_{g\text{-fractal}}$ (nm)	Intermixing domain size ξ (nm)
PTzBI-*d*F:BTP-Br	Fresh	23	40
	Aged	36	47
PTzBI-*d*F:BTP-TBr	Fresh	32	42
	Aged	40	85
