# Supplementary material for: Importance of structural hinderance in performance–stability equilibrium of organic photovoltaics
Source: Nat Commun. 2022 Oct 8;13:5946. doi: 10.1038/s41467-022-33754-3 (PMC9547926; doi:10.1038/s41467-022-33754-3)
Supplement: Supplementary file 2 — Reporting Summary [file 41467_2022_33754_MOESM2_ESM.pdf]

nature research

Corresponding author(s): Alex K.-Y. Jen

Solar Cells Reporting Summary

Nature Research wishes to improve the reproducibility of the work that we publish. This form is intended for publication with all accepted papers reporting the characterization of photovoltaic devices and provides structure for consistency and transparency in reporting. Some list items might not apply to an individual manuscript, but all fields must be completed for clarity.

For further information on Nature Research policies, including our data availability policy, see Authors & Referees.

Experimental design

Please check: are the following details reported in the manuscript?

1. Dimensions

Item	Yes/No	Response
Area of the tested solar cells	☒ Yes	The device area is 0.0414 cm2 (see "Methods").
	☐ No	*Explain why this information is not reported/not relevant.*
Method used to determine the device area	☒ Yes	Image measuring instrument.
	☐ No	*Explain why this information is not reported/not relevant.*

2. Current-voltage characterization

Item	Yes/No	Response
Current density-voltage (J-V) plots in both forward and backward direction	☐ Yes	*State where this information can be found in the text.*
	☒ No	Hysteresis is not observed in organic solar cells, thus only forward scan was applied.
Voltage scan conditions *For instance: scan direction, speed, dwell times*	☒ Yes	We used a dwell time of 20 ms for each voltage and a step voltage of 0.02 V (forward).
	☐ No	*Explain why this information is not reported/not relevant.*
Test environment *For instance: characterization temperature, in air or in glove box*	☒ Yes	The cells were measured in N2-filled grove box with temperature around 25 °C during I-V scans.
	☐ No	*Explain why this information is not reported/not relevant.*
Protocol for preconditioning of the device before its characterization	☐ Yes	No preconditioning protocol like encapsulation was used before characterization.
	☒ No	No preconditioning protocol was applied before characterization.
Stability of the J-V characteristic *Verified with time evolution of the maximum power point or with the photocurrent at maximum power point; see ref. 7 for details.*	☒ Yes	See MPP tracking in Supplementary Fig. 26.
	☐ No	N.A.

3. Hysteresis or any other unusual behaviour

Item	Yes/No	Response
Description of the unusual behaviour observed during the characterization	☒ Yes	No hysteresis or any other unusual behaviors were observed in the testing.
	☐ No	No hysteresis or any other unusual behaviors were observed in the testing.
Related experimental data	☐ Yes	*State where this information can be found in the text.*
	☒ No	N.A.

4. Efficiency

Item	Yes/No	Response
External quantum efficiency (EQE) or incident photons to current efficiency (IPCE)	☒ Yes	Fig. 2b, Supplementary Fig. 13b, and Supplementary Fig. 27a.
	☐ No	*Explain why this information is not reported/not relevant.*
A comparison between the integrated response under the standard reference spectrum and the response measure under the simulator	☒ Yes	Supplementary Fig. 13b, and Supplementary Fig. 14a.
	☐ No	*Explain why this information is not reported/not relevant.*
For tandem solar cells, the bias illumination and bias voltage used for each subcell	☐ Yes	*State where this information can be found in the text.*
	☒ No	N.A.

5. Calibration

Light source and reference cell or sensor used for the characterization

☒ Yes ☐ No

A class AAA solar simulator (Enlitech SS-F5) was used as light source, providing 100 mW cm-2 of simulated AM I.5G irradiation, which was calibrated by a standard silicon solar cell (KG2 filter).

Explain why this information is not reported/not relevant.

Confirmation that the reference cell was calibrated and certified

☒ Yes ☐ No

The reference cell is calibrated through tracing to NREL or ISE Fraunhofer.

Explain why this information is not reported/not relevant.

Calculation of spectral mismatch between the reference cell and the devices under test

☐ Yes ☒ No

N.A.

Spectral mismatch was not calculated in this system.

6. Mask/aperture

Size of the mask/aperture used during testing

☒ Yes ☐ No

The aperture area of 0.033 cm2 were used for testing cells with area of 0.0414 cm2.

Explain why this information is not reported/not relevant.

Variation of the measured short-circuit current density with the mask/aperture area

☐ Yes ☒ No

State where this information can be found in the text.

No evident Jsc variation is observed when using mask with different area.

7. Performance certification

Identity of the independent certification laboratory that confirmed the photovoltaic performance

☐ Yes ☒ No

State where this information can be found in the text.

We focus on the structure-stability relationship in this work.

A copy of any certificate(s)
Provide in Supplementary Information

☐ Yes ☒ No

State where this information can be found in the text.

N.A.

8. Statistics

Number of solar cells tested

☒ Yes ☐ No

We tested at least 16 cells for each type of devices, with the standard error shown in Table 1.

Explain why this information is not reported/not relevant.

Statistical analysis of the device performance

☒ Yes ☐ No

Statistical analysis of the efficiency evolution shown in Supplementary Fig. 26 (see the error bar).

N.A.

9. Long-term stability analysis

Type of analysis, bias conditions and environmental conditions
For instance: illumination type, temperature, atmosphere humidity, encapsulation method, preconditioning temperature

☒ Yes ☐ No

Thermal stability (Fig. 3c, d and supplementary Fig. 22, 23, 24); light stability (supplementary Fig. 26).

N.A.
